# Cebulactam A_3_, a Macrolactam from Marine-Derived *Saccharopolyspora* sp. PG10, and Its Antibacterial Activity

**DOI:** 10.3390/md24060211

**Published:** 2026-06-14

**Authors:** Chan Kim, Thinh T. M. Bui, Hyeongju Jeong, Soohyun Um, Kyuho Moon

**Affiliations:** 1 Department of Integrated Drug Development and Natural Products, Graduate School, Kyung Hee University, Seoul 02447, Republic of Korea; rjsdl5@khu.ac.kr (C.K.);; 2 College of Pharmacy and Institute of Integrated Pharmaceutical Sciences, Kyung Hee University, Seoul 02447, Republic of Korea; hjjeong@khu.ac.kr; 3 Department of Forest Products and Biotechnology, Kookmin University, Seoul 02707, Republic of Korea

**Keywords:** marine sediment, secondary metabolite, structural elucidation, antibacterial activity

## Abstract

Chemical analysis of the marine-derived *Saccharopolyspora* sp. PG10 led to the isolation of a novel macrolactam, cebulactam A_3_ (**1**), along with four known congeners, cebulactams A_1_ and A_2_ (**2** and **3**) and shengliangmycins B and D (**4** and **5**). The structure of **1** was established by high-resolution mass spectrometry (HRMS) and comprehensive nuclear magnetic resonance (NMR) analyses, and its absolute configuration was determined using Mosher’s method. Genome analysis identified a putative biosynthetic gene cluster consistent with a hybrid polyketide pathway. Antimicrobial evaluation revealed that shengliangmycin B exhibited the strongest activity, whereas cebulactam analogs exhibited weaker effects. These findings expand the structural diversity of cebulactam-type macrolactams and provide insights into their stereochemical variation.

## 1. Introduction

Marine-derived microorganisms have emerged as an important source of structurally diverse secondary metabolites, many of which exhibit biological activities not readily observed in compounds from terrestrial origins [1,2]. The physicochemical constraints of marine environments, including osmotic stress, nutrient limitation, and intense ecological competition, are thought to drive the evolution of specialized metabolic pathways [3]. Consequently, marine-derived actinomycetes frequently produce polyketides and peptide-derived metabolites with unusual scaffolds, oxidative patterns, and stereochemical complexity [4,5,6]. Although substantial progress has been made over the past decades, the chemical space represented by marine actinomycetes remains incompletely explored, particularly at the level of strain-specific metabolite diversity [7,8].

Among marine actinomycetes, members of the genus *Saccharopolyspora* have been recognized as prolific producers of bioactive natural products. These species produce a wide range of secondary metabolites, including macrolides, polyketides, and hybrid structures, many of which exhibit antibacterial or cytotoxic activities [9,10,11]. Notably, *Saccharopolyspora*-derived compounds are often derived from modular polyketide synthase (PKS) systems and exhibit extensive post-assembly tailoring, resulting in structurally complex frameworks [12,13]. However, compared with other well-studied actinomycete genera, such as *Streptomyces*, the metabolic output and biosynthetic potential of marine-derived *Saccharopolyspora* strains remain relatively unknown [11]. Hence, targeted exploration of this genus may yield novel chemical scaffolds or structural variants of known metabolite families.

Macrolactams constitute a distinct class of nitrogen-containing macrocyclic natural products that are frequently assembled through hybrid polyketide synthase–nonribosomal peptide synthetase (PKS–NRPS) or PKS-like biosynthetic pathways. These compounds are characterized by large ring systems incorporating amide functionalities, often appended with aromatic moieties and multiple oxygenated stereocenters [14,15]. They exhibit a range of biological activities, including antibacterial and antiproliferative effects, and have attracted interest as potential leads for drug discovery [16]. Structurally, macrolactams exhibit significant diversity in ring size, substitution pattern, and stereochemistry, reflecting the flexibility of their biosynthetic machinery. In particular, subtle changes in stereochemistry or oxidation state can give rise to distinct analogs within a single metabolite family.

The cebulactam family represents a group of macrolactam natural products derived from *Saccharopolyspora* species [17]. These compounds are biosynthetically related to ansamycin-type scaffolds and are thought to originate from 3-amino-5-hydroxybenzoic acid (AHBA) starter units extended by polyketide elongation [18,19]. Prior studies have identified several cebulactam analogs with closely related core structures but different substitution patterns and stereochemical features. However, the structural diversity within this family remains limited. Moreover, the relationship between stereochemical variation and biological activity has not been fully explored. In particular, epimeric variation at specific stereocenters has not been extensively documented, leaving open questions about the origin and functional effects of such diversity.

Integration of genomic information has become an important approach in natural product research, particularly for linking chemical structures to their biosynthetic origins [20]. In macrolactam-producing actinomycetes, biosynthetic gene clusters (BGCs) typically encode modular PKS systems and enzymes responsible for the formation of AHBA-derived starter units. Such information can support biosynthetic proposals and help rationalize structural variations within related compound families.

**Figure 1:**
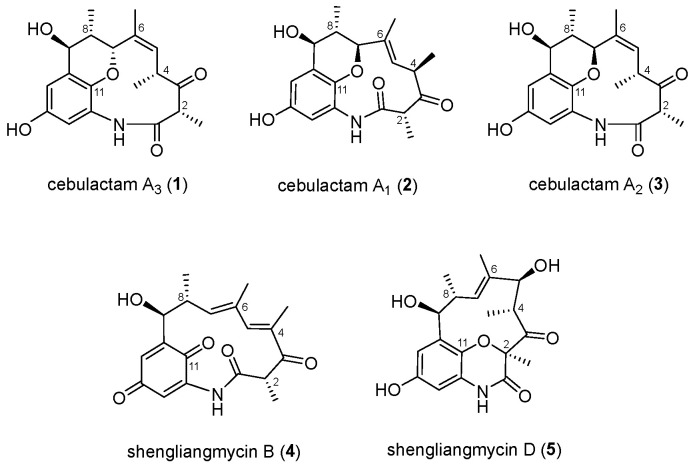
Structures of cebulactam and shengliangmycin analogs.

As part of our efforts to explore metabolite diversity in marine-derived actinomycetes, we investigated a *Saccharopolyspora* strain (PG10) isolated from marine sediment in Busan, Republic of Korea. Chemical analysis led to the isolation of a previously unreported macrolactam, cebulactam A_3_ (**1**), together with four known congeners (Figure 1, Table S1). Structural and stereochemical analyses revealed that **1** is a C-7 epimer of a previously reported analog, and genome analysis identified a putative BGC consistent with its assembly.

## 2. Results and Discussion

### 2.1. Isolation of Strains Using Diverse Culture Media

In total, 21 strains were isolated from marine sediment using five different media under two antibiotic conditions (Table S2). Among these, strains obtained under antibiotic set A were more abundant, with TWYE medium yielding the highest number of isolates. Of the isolated strains, only strains PG1–PG11 were successfully cultivated in liquid medium and preserved for further analysis. LC/MS-based screening of these cultures identified strain PG10 as the most promising candidate for chemical analysis. Phylogenetic analysis based on 16S rRNA gene sequencing subsequently identified this strain as a member of the genus *Saccharopolyspora*.

### 2.2. Structural Elucidation

Cebulactam A_3_ (**1**) was isolated as a brown powder. Its molecular formula was established as C_19_H_23_NO_5_ from HR-ESI-MS *m*/*z* 346.1644 [M + H]^+^ (calculated for C_19_H_24_NO_5_, *m*/*z* 346.1649), indicating nine degrees of unsaturation. The structure elucidation was carried out using 1D and 2D NMR techniques, including correlation spectroscopy (COSY), heteronuclear single quantum correlation (HSQC), heteronuclear multiple bond correlation (HMBC), and rotating-frame Overhauser effect spectroscopy (ROESY), along with infrared spectroscopy (IR) and mass spectrometry (MS).

The ^1^H and ^13^C NMR spectra (Table 1) indicated the presence of a highly functionalized macrolactam framework bearing an aromatic moiety. The ^13^C NMR spectrum exhibited signals for two carbonyl carbons, namely an amide carbonyl (*δ*_C_ 169.8, C-1) and a ketone carbonyl (*δ*_C_ 209.2, C-3). The aromatic region exhibited signals characteristic of a 1,3,4,5-tetrasubstituted benzene ring, supported by two proton signals at *δ*_H_ 6.36 (d, *J* = 3.0 Hz, H-13) and 6.82 (d, *J* = 3.0 Hz, H-15). In the olefinic region, one olefinic proton and two olefinic carbons (*δ*_C_ 130.0 and 135.1) were observed, indicating the presence of a double bond. In addition, four methyl groups and two oxygenated methines suggested a highly substituted and branched carbon skeleton.

**Figure 2:**
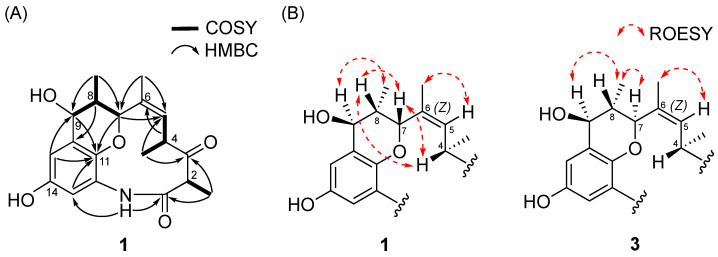
(**A**) Key COSY and HMBCs of **1**. (**B**) Key ROESY correlations of **1** and **3**.

Detailed analysis of the COSY spectrum established three substructures: fragment I (C-2/2-Me), fragment II (4-Me/C-4/C-5), and fragment III (C-7/C-8/C-9/8-Me) (Figure 2A). These fragments were subsequently assembled through key HMBCs. Correlations from 2-Me (*δ*_H_ 1.10) to C-1 (*δ*_C_ 169.8) and C-3 (*δ*_C_ 209.2) established a methylmalonyl moiety, which was extended to C-5 (*δ*_C_ 130.0) based on HMBC correlations from both 2-Me and 4-Me (*δ*_H_ 1.06) to C-3. Further extension of the carbon framework to C-9 (*δ*_C_ 69.1) was achieved by incorporation of fragment III, supported by HMBC correlations from 6-Me (*δ*_H_ 1.77) to C-5 and C-7 (*δ*_C_ 80.9) and from H-7 (*δ*_H_ 4.16) to C-5. The side chain was connected to a tetrasubstituted aromatic ring at C-10 (*δ*_C_ 129.1), as evidenced by HMBC correlations from H-8 (*δ*_H_ 2.05) to C-10, H-9 (*δ*_H_ 4.20) to C-11 (*δ*_C_ 140.1), and H-15 (*δ*_H_ 6.82) to C-9 (*δ*_C_ 69.1). Closure of the 13-membered macrolactam ring was established by HMBC correlations from the amide proton (*δ*_H_ 8.87) to C-1, C-12 (*δ*_C_ 125.2), and C-13 (*δ*_C_ 112.8). Eight of the nine degrees of unsaturation were accounted for by two carbonyl groups, one benzene ring, one olefinic bond, and the macrolactam ring. The remaining degree of unsaturation was attributed to an additional ring formed via an ether bridge between C-7 and C-11, supported by key HMBCs from H-7 to C-11. Accordingly, **1** was identified as a tricyclic macrolactam.

The geometry of the double bond was assigned as *Z*, supported by the ROESY correlations between 6-Me and H-5 (*δ*_H_ 5.33) and between H-7 and H-4 (*δ*_H_ 3.79). The planar structure of **1** was identical to that of cebulactam A_2_ (**3**). However, noticeable differences in the chemical shifts around C-7 suggested that **1** is a diastereomer of **3**. The relative configuration of **1** was established by comprehensive analysis of the ROESY spectrum (Figure 2B). In particular, the ROESY correlation between H-9 and 8-Me (*δ*_H_ 1.05) indicated an *anti* relationship between 8-Me and 9-OH. Additionally, the ROESY correlation between H-7 and H-8 suggested that these protons are *syn*-oriented. Furthermore, the ROESY correlation between H-8 and H-4 indicated that 8-Me and 4-Me are located on the same face of the molecule. The configuration at C-2 was tentatively assigned as *S* based on biosynthetic considerations. This assignment was supported by the co-produced congeners cebulactams A_1_ and A_2_ and shengliangmycin B, which possess the same *S* configuration at the corresponding C-2 position. Moreover, previous reports of structurally related congeners bearing the same C-2 configuration further support this assignment [19,21]. Collectively, the ROESY data and biosynthetic considerations supported the assignment of the relative configuration of **1** as 2S*, 4R*, 7R*, 8S*, 9S*.

**Figure 3:**
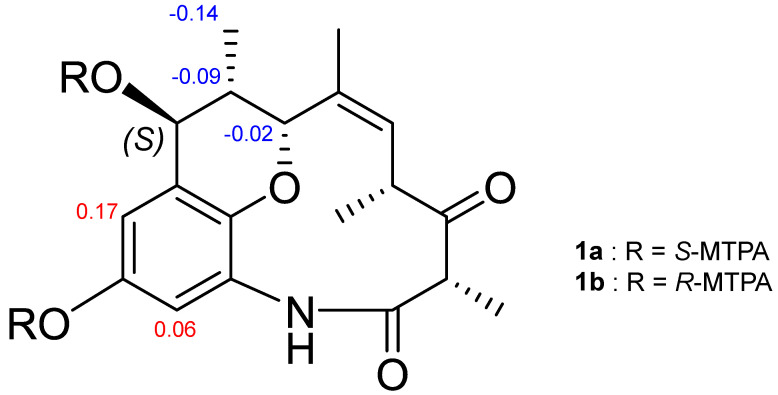
Δ*δ_S_*_−*R*_ values (ppm) obtained for *S*- and *R*-MTPA esters (**1a** and **1b**, respectively).

The absolute configuration at C-9 was determined using the modified Mosher’s method [22,23,24]. The secondary hydroxy group was derivatized with (*R*)- and (*S*)-MTPA chloride to afford the corresponding diastereomeric Mosher esters, which were then analyzed by ^1^H NMR spectroscopy. Interpretation of the Δ*δ_S−R_* values led to the assignment of the absolute configuration at C-9 as *S*. Combined with the aforementioned relative stereochemical analysis, the absolute configuration of **1** was assigned as *2S*, *4R*, *7R*, *8S*, *9S* (Figure 3). Thus, **1** was identified as the C-7 epimer of cebulactam A_2_ (**3**) and was designated as cebulactam A_3_ (**1**). The distinct CD profiles of cebulactam A_3_ (**1**) and cebulactam A_2_ (**3**), which showed different Cotton effect patterns below 250 nm, were consistent with this epimeric relationship (Figure S58).

By comparison of their NMR spectroscopic data with those reported in the literature, compounds **2**–**5** were identified as cebulactam A_1_ [17], cebulactam A_2_ [17], shengliangmycin B [19], and shengliangmycin D [19], respectively.

Bioinformatic analysis of the draft genome of strain PG10 identified a putative cebulactam biosynthetic gene cluster (BGC) spanning approximately 60 kb, containing 40 predicted coding sequences (CDSs) (Table S3). This cluster was found to encode a hybrid type I PKS system along with enzymes potentially involved in precursor supply, tailoring, and regulation, consistent with the biosynthesis of macrolactam-type polyketides.

**Figure 4:**
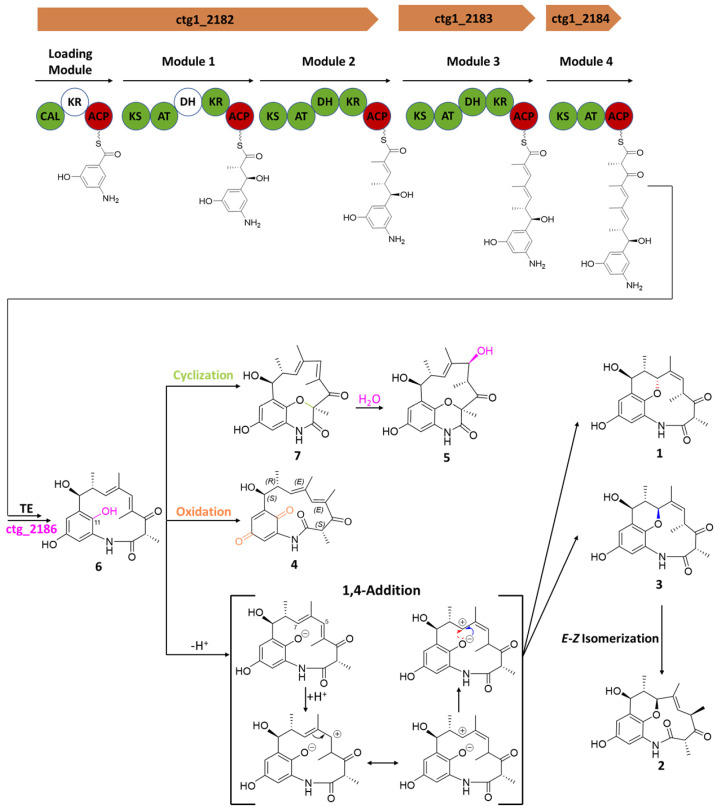
Proposed biosynthetic pathway for cebulactam analogs (**1**–**5**). CAL = CoA ligase, ACP = acyl carrier protein, KS = ketosynthase, AT = acyltransferase, KR = ketoreductase, and DH = dehydratase.

The PKS modules (ctg1_2182–2184) were proposed to utilize AHBA as the starter unit and methylmalonyl-CoA and malonyl-CoA as extender units to construct the macrolactam backbone **6**. The presence of CDSs associated with AHBA biosynthesis further supported this assignment. Subsequently, **6** undergoes cyclization between C-2 and the 11-OH group, followed by hydroxylation, to afford **5**. In parallel, the *p*-dihydroxybenzene moiety is oxidized to a 1,4-benzoquinone, yielding **4**. Further cyclization between C-7 and C-11 via an ether bridge generates **1** and **3**, which exist as a pair of epimers. Finally, **3** undergoes isomerization of the double bond to form **2** (Figure 4).

The formation of the C-7 epimeric pair can be alternatively explained by the intramolecular 1,4-addition via an allylic carbocation intermediate. First, activation of the conjugated diene generates an allylic carbocation, with the positive charge delocalized over the C-4/C-7 framework. Subsequently, the 11-OH can act as an intramolecular nucleophile and attack the planar allylic cation at C-7 from either the *re*- or *si*-face. This non-stereoselective intramolecular capture leads to the formation of two epimeric products, cebulactams A_3_ (**1**) and A_2_ (**3**), thereby accounting for the observed stereochemical variation at C-7.

### 2.3. Antimicrobial Activity Assessment

The antimicrobial activities of the isolated compounds were evaluated against five microbial strains, including the Gram-positive bacterium *Bacillus subtilis*, the Gram-negative bacteria *Pseudomonas aeruginosa*, *Escherichia coli*, and *Erwinia rhapontici*, and the fungus *Candida albicans*, using a broth microdilution assay. Among the tested strains, inhibitory activity was only detected against *B. subtilis* and *P. aeruginosa*, whereas no activity was detected against *E. coli*, *Er. rhapontici*, or *C*. *albicans* under the tested conditions. Among the tested compounds, shengliangmycin B exhibited the most potent activity, with IC_50_ values of 3.37 µM against *P. aeruginosa* and 14.10 µM against *B. subtilis*, whereas shengliangmycin D exhibited no detectable activity. In contrast, cebulactam derivatives exhibited only weak to moderate activity against *P. aeruginosa*; the IC_50_ values for cebulactams A_1_ (**2**), A_2_ (**3**), and A_3_ (**1**) were 200.50, 218.98, and 203.07 µM, respectively (Table S4). Notably, cebulactam A_2_ was the only analog that exhibited measurable activity against *B. subtilis* (IC_50_ = 109.67 µM). These results indicate that shengliangmycin-type compounds are more potent than cebulactam analogs and that variation at C-7 has a limited impact on antibacterial activity within this scaffold.

## 3. Materials and Methods

### 3.1. General Experimental Procedures

Optical rotations were measured using a JASCO P-2000 polarimeter (JASCO, Hachioji, Tokyo, Japan) in a 1.0 cm cell at room temperature. IR spectra were recorded on a PerkinElmer Spectrum 400 FT-IR/FT-NIR spectrometer (PerkinElmer, Waltham, MA, USA). CD spectra were obtained with a 1 mm cell using Chirascan Plus (Applied Photophysics Ltd., Leatherhead, Surrey, UK). ^1^H, ^13^C, and 2D NMR spectra were acquired on Bruker Avance II 600 and 700 MHz NMR spectrometers (Bruker, Billerica, MA, USA) at the Korea Basic Science Institute (KBSI), Ochang, Republic of Korea; MTPA ester derivatives were analyzed on the 600 MHz instrument. UV spectrometry and low-resolution electrospray ionization mass spectrometry (LR-ESI-MS) were performed using an Agilent G6125B MSD coupled to an Agilent 1260 Infinity II LC system (Agilent Technologies, Santa Clara, CA, USA) equipped with a Phenomenex Luna reversed-phase C_18_ column (100 × 4.6 mm, 5 μm). LC/MS analysis was performed using H_2_O (Daejung Chemicals & Metals, Siheung, Republic of Korea) and CH_3_CN (Daejung Chemicals & Metals, Siheung, Republic of Korea) as mobile phases, each containing 0.1% formic acid (Sigma-Aldrich, St. Louis, MO, USA), at a flow rate of 0.4 mL/min. The gradient was as follows: 10% CH_3_CN for 3 min, 10–100% CH_3_CN over 20 min, 100% CH_3_CN for 5 min. HR-ESI-MS data were recorded on an Agilent 1290 Series HPLC system coupled to an Agilent 6530 iFunnel Q-TOF mass spectrometer (Agilent Technologies, Santa Clara, CA, USA). Microplate absorbance was measured using an Epoch microplate spectrophotometer (BioTek Instruments, Winooski, VT, USA).

### 3.2. Bacterial Material

#### 3.2.1. Bacterial Isolation and Identification

Strain PG10 was isolated in August 2024 from marine sediment collected from Gwangalli, Busan, Republic of Korea (35°09′19″ N, 129°08′16″ E, depth: 13 m). The sediment samples were dried in a clean bench for 2 days. The dried sediment (2 g) was then mixed with distilled water (12 mL) at a 1:6 ratio and sonicated for 20 min. Subsequently, 100 μL of the supernatant was spread onto prepared SIM, ISP5, SCNA, TWYE, and GS agar plates (for details, see Table S5). All media were prepared using artificial seawater containing 33 g/L sea salt to approximate the salinity of natural seawater. Depending on the medium, antibiotic set A [cycloheximide (100 mg/L; Thermo Scientific, Waltham, MA, USA) and nalidixic acid (20 mg/L; Acros Organics, Geel, Belgium)] or antibiotic set B [cycloheximide (80 mg/L), gentamicin (10 mg/L; Thermo Scientific, Waltham, MA, USA), and novobiocin (10 mg/L; Thermo Scientific, Waltham, MA, USA)] was added. Strain PG10 was then purified by cultivation on TWYE agar. To identify strain PG10 at the molecular level, its 16S rRNA gene was sequenced using two independent primers: 785F and 907R. The resulting forward and reverse reads were assembled based on their overlapping region to generate a single contig of 1458 bp. BLASTN analysis [25] of the assembled sequence revealed the highest similarity to *S. cebuensis* strain SPE 10-1 (accession no. NR_044047.1), with 1448 identical nucleotides out of 1452 aligned positions, corresponding to 99.72% sequence identity. Thus, strain PG10 was assigned to the genus *Saccharopolyspora* and was most closely related to *S. cebuensis* (Figure S59).

#### 3.2.2. Whole-Genome Sequencing and Assembly

Whole-genome sequencing was performed by Macrogen, Inc. (Seoul, Republic of Korea). Genomic DNA of *Saccharopolyspora* sp. PG10 was subjected to de novo whole-genome sequencing using a hybrid strategy combining Illumina short-read and PacBio SMRT sequencing. The genome was assembled using the Microbial Genome Analysis pipeline (SMRTLINK, v25.1.0.257715) and subsequently polished with Illumina reads. The final assembly comprised two contigs with a total length of 6.29 Mb, an N50 value of 6.24 Mb, and a GC content of 72.7%. The estimated genome size was 6.01 Mb, and the average read depth was 114.37-fold. Assembly validation metrics indicated high quality, including 99.32% Illumina read mapping with 100% coverage, 99.74% PacBio read mapping with 100% coverage, and 100% complete BUSCO recovery, supporting the high completeness and reliability of the assembled genome. Moreover, genome-based phylogenomic analysis using the TYGS platform [26] placed strain PG10 in a clade with *S. cebuensis* JCM 18116, indicating a close phylogenomic relationship between the two strains (Figure S60). Together with the 16S rRNA gene sequence data, these results support the assignment of strain PG10 as a member of the genus *Saccharopolyspora* that is most closely related to *S. cebuensis*. The whole-genome shotgun project has been deposited in the NCBI database under the accession JBWXRK000000000. The associated BioProject and BioSample accession numbers are PRJNA1448979 and SAMN57104874, respectively.

### 3.3. Cultivation and Extraction

Strain PG10 was initially cultivated in 50 mL of TSBY seawater medium (Table S5) in a 100 mL Erlenmeyer flask. After incubation at 27 °C for 3 days on a rotary shaker at 180 rpm, 3.5 mL of the seed culture was transferred into a 500 mL Erlenmeyer flask containing 150 mL of R4 seawater medium and further cultivated for 2 days under the same conditions. Subsequently, 20 mL of this culture was inoculated into 1 L of R4 seawater medium (Table S5) in a 2.5 L Ultra Yield flask and fermented at 27 °C for 5 days with shaking at 180 rpm. The entire culture broth was then extracted with ethyl acetate (EtOAc). The organic layer was separated using a 3 L separatory funnel, dried over anhydrous Na_2_SO_4_, and concentrated *in vacuo* to yield 1.7 g of dried extract.

### 3.4. Purification of Cebulactams and Shengliangmycins

The dried extract of strain PG10 was fractionated by reversed-phase C_18_ open-column chromatography (YMC ODS-A-C_18_, 50 μm) using 300 mL each of aqueous MeOH (20%, 40%, 60%, 80%, and 100%), followed by MeOH/DCM (1:1). From the 40% aqueous MeOH fraction, cebulactams A_1_–A_3_ and shengliangmycin D were isolated by semi-preparative reversed-phase HPLC (YMC-Pack ODS-A-C_18_ column, 5 μm, 250 × 10 mm) using 20% aqueous CH_3_CN containing 0.1% formic acid at a flow rate of 2.0 mL/min, with UV detection at 280 nm. As a result, compounds **1**, **2**, **3**, and **5** were obtained as pure compounds with the following retention times and yields: **1** (t_R_ = 25 min, 15.0 mg), **2** (t_R_ = 22 min, 19.6 mg), **3** (t_R_ = 39 min, 28.3 mg), and **5** (t_R_ = 18 min, 16.5 mg). From the 60% aqueous MeOH fraction, shengliangmycin B was purified using the same HPLC method with 33% aqueous CH_3_CN containing 0.1% formic acid, affording compound **4** as a pure compound (t_R_ = 30 min, 8.3 mg).

Cebulactam A_3_ (**1**):

Brown powder; ${\left[\alpha \right]}_{D}^{25}$ −125.7 (*c* 0.10, MeOH); IR *ν*_max_ (ATR) 3245, 2974, 2932, 1714, 1661, 1607, 1461, 1376, 1198, 1155, 1005 cm^−1^; UV (MeOH) λ_max_ (log ε) 200 (2.24), 305 (1.54) nm; HR-ESI-MS [M + H]^+^ *m*/*z* 346.1644 (calculated for C_19_H_24_NO_5_ 346.1649) (Figures S55–S57); ^1^H NMR (DMSO-*d*_6_, 700 MHz) and ^13^C NMR (DMSO-*d*_6_, 175 MHz) (Figures S1–S10).

### 3.5. MTPA Esterification of Cebulactam A_3_

Two samples of cebulactam A_3_ (1.5 mg each) were placed in separate 40 mL vials and lyophilized overnight. Each sample was treated with anhydrous pyridine (1.0 mL) and DMAP (1.0 mg) under nitrogen and stirred at room temperature for 5 min. *R*- or *S*-MTPA-Cl (30 µL) was added to each vial, and the mixtures were stirred at 35 °C for 4 h. Purification by reversed-phase HPLC on a YMC-Pack C_8_ column (5 µm, 250 × 10.0 mm) using a stepwise CH_3_CN/H_2_O gradient (60–100% CH_3_CN over 60 min, followed by 100% CH_3_CN for 20 min; 2 mL/min; UV 280 nm) yielded the *S*-MTPA ester of cebulactam A_3_ (**1a**) and the *R*-MTPA ester (**1b**), both of which eluted at 40 min. The Δ*δ_S−R_* values were determined based on the ^1^H NMR and ^1^H − ^1^H COSY spectra.

*S*-MTPA ester of cebulactam A_3_ (**1a**): ^1^H NMR (600 MHz, DMSO-*d*_6_) *δ*_H_ 9.12 (NH, s), 7.46–7.58 (12H, overlapped), 7.06 (1H, d, *J* = 2.0), 6.79 (1H, d, *J* = 2.0), 6.23 (1H, d, *J* = 9.5), 5.30 (1H, d, *J* = 8.5), 4.61 (1H, d, *J* = 10.0), 3.85 (2H, overlapped), 3.59 (3H, s), 3.46 (3H, s), 2.59 (1H, m), 1.70 (3H, s), 1.12 (3H, d, *J* = 6.5), 1.03 (3H, d, *J* = 6.5), 0.86 (3H, d, *J* = 6.5); LR-ESI-MS [M + Na]^+^ *m*/*z* 800.1.

*R*-MTPA ester of cebulactam A_3_ (**1b**): ^1^H NMR (600 MHz, DMSO-*d*_6_) *δ*_H_ 9.11 (NH, s), 7.46–7.58 (16H, overlapped), 7.00 (1H, d, *J* = 2.0), 6.62 (1H, d, *J* = 2.0), 6.25 (1H, d, *J* = 9.5), 5.35 (1H, d, *J* = 9.0), 4.63 (1H, d, *J* = 10.0), 3.81 (1H, m), 3.78 (1H, q, *J* = 6.5), 3.55 (3H, s), 3.53 (3H, s), 2.68 (1H, m), 1.77 (3H, s), 1.09 (3H, d, *J* = 6.5), 1.01 (3H, d, *J* = 6.5), 1.00 (3H, d, *J* = 6.5); LR-ESI-MS [M + Na]^+^ *m*/*z* 800.1.

### 3.6. Antimicrobial Activity Assay

The Gram-positive bacterium *B. subtilis* (ATCC 6051) and the Gram-negative bacteria *P. aeruginosa* (KCTC 22073), *E. coli* (ATCC 11775), and *Er. rhapontici* (ATCC 29283) were cultured on Luria–Bertani agar (LB agar), while the fungus *C. albicans* (KCTC 7965) was cultured on yeast extract–peptone–dextrose agar (YPD agar). After overnight incubation at 27 °C, bacterial cells were transferred to LB broth and cultured at 30 °C for 24 h, whereas *C. albicans* was cultured in YPD broth under the same conditions. The harvested bacterial and fungal cells were inoculated into Mueller–Hinton broth (MHB) and YPD broth, respectively, to an initial optical density (OD) of 0.0008 at 600 nm. Test compounds were dissolved in DMSO and serially diluted two-fold with the corresponding broth to final concentrations of 200–0.4 µg/mL. The plates were then incubated at 30 °C for 18 h, and microbial growth was measured by recording the absorbance at 600 nm. Gentamicin and cycloheximide were used as reference compounds for bacteria and *C. albicans*, respectively, and were tested under the same conditions at final concentrations of 200–0.4 µg/mL.

## 4. Conclusions

In this study, a previously undescribed macrolactam, cebulactam A_3_ (**1**), was isolated from the marine-derived *Saccharopolyspora* sp. PG10, together with four known congeners. The structure of **1** was elucidated by comprehensive spectroscopic analyses, and its absolute configuration was established using Mosher’s method. ROESY data identified **1** to be a C-7 epimer of cebulactam A_2_, representing a rare example of stereochemical variation within the cebulactam family. Genome analysis identified a putative BGC consistent with a hybrid PKS pathway, and a plausible mechanism was proposed to account for the formation of the C-7 epimeric pair.

Antibacterial evaluation provided additional biological information on the isolated analogs, including the previously unreported antibacterial activity of shengliangmycin B under the tested conditions. Overall, these findings expand the structural diversity of cebulactam-type macrolactams and provide insights into their stereochemical and biosynthetic variability.

## Figures and Tables

**Table 1:** ^1^H and ^13^C NMR spectroscopic data of cebulactam A_3_ (**1**) in DMSO-*d*_6_.

Position	Cebulactam A_3_ (1)	
	*δ*_C_, Type	*δ*_H_, Mult (*J* in Hz)
NH	-	8.87, s
1	169.8, C	-
2	48.4, CH	3.75, q (6.5)
2-Me	17.1, CH_3_	1.10, d (6.5)
3	209.2, C	-
4	45.5, CH	3.79, m
4-Me	19.5, CH_3_	1.06, d (6.5)
5	130.0, CH	5.33, dd (9.0, 1.0)
6	135.1, C	-
6-Me	24.7, CH_3_	1.77, d (1.0)
7	80.9, CH	4.16, d (11.0)
8	38.7, CH	2.05, m
8-Me	13.9, CH_3_	1.05, d (6.5)
9	69.1, CH	4.20, m
10	129.1, C	-
11	140.1, C	-
12	125.2, C	-
13	112.8, CH	6.36, d (3.0)
14	150.3, C	-
15	111.7, CH	6.82, d (3.0)

^1^H and ^13^C data were recorded at 700 and 175 MHz, respectively.

## Data Availability

The original contributions presented in this study are included in the article.

## Supplementary Materials

The following supporting information can be downloaded at: https://www.mdpi.com/article/10.3390/md24060211/s1, Figures S1–S10: NMR spectra of cebulactam A_3_ (**1**); Figures S11–S14: NMR spectra of MTPA esters of cebulactam A_3_ (**1**); Figures S15–S24: NMR spectra of cebulactam A_1_ (**2**); Figures S25–S34: NMR spectra of cebulactam A_2_ (**3**); Figures S35–S44: NMR spectra of shengliangmycin B (**4**); Figures S45–S54: NMR spectra of shengliangmycin D (**5**); Figure S55: UV spectrum of cebulactam A_3_ (**1**); Figure S56: HR-ESI-MS data of cebulactam A_3_ (**1**); Figure S57: FT-IR spectrum of cebulactam A_3_ (**1**); Figure S58: The circular dichroism (CD) spectra of cebulactam A_3_ and A_2_ (**1** and **3**) at a concentration of 0.1 mg/mL in MeOH; Figure S59: 16S rRNA gene sequence data of PG10; Figure S60: Whole-genome-based phylogenetic tree of PG10; Table S1: ^1^H and ^13^C NMR spectroscopic data of compounds **2**–**5** in DMSO-*d*_6_; Table S2: Isolated strains from diverse media; Table S3: Predicted functions of CDSs in the cebulactam biosynthetic gene cluster based on whole-genome sequencing data; Table S4: Growth-inhibitory activities (IC_50_) of compounds **1**–**5** against the tested fungal and bacterial strains; Table S5: Isolation and cultivation media; Supplementary note.
