# Supplementary material for: Cebulactam A3, a Macrolactam from Marine-Derived Saccharopolyspora sp. PG10, and Its Antibacterial Activity
Source: Mar Drugs. 2026 Jun 14;24(6):211. doi: 10.3390/md24060211 (PMC13302746; doi:10.3390/md24060211)
Supplement: Supplementary file 1 [file marinedrugs-24-00211-s001.zip › marinedrugs-4344129-supplementary.pdf]

*Supporting information for*

# *Cebulactam A$_3$, a Macrolactam from Marine-Derived Saccharopolyspora* sp. PG10*, and Its Antibacterial Activity*

*Chan Kim[1,†], Thinh T. M. Bui[1,†], Hyeongju Jeong[2,†], Soohyun Um[3]* and Kyuho Moon[1,2]**

*[1]Department of Integrated Drug Development and Natural Products, Graduate School, Kyung Hee University, Seoul 02447, Republic of Korea*

*[2]College of Pharmacy and Institute of Integrated Pharmaceutical Sciences, Kyung Hee University, Seoul 02447, Republic of Korea*

*[3]Department of Forest Products and Biotechnology, Kookmin University, Seoul 02707, Republic of Korea*

**Authors to whom correspondence should be addressed.*

*[†]These authors contributed equally to this work.*

# Table of contents

**Figure S1.** $^1$H-NMR spectrum of cebulactam $A_3$ (**1**) at 700 MHz in DMSO-$d_6$.

**Figure S2.** Expanded $^1$H-NMR spectrum ($\delta_H$ 6.9–6.3) of cebulactam $A_3$ (**1**) at 700 MHz in DMSO-$d_6$.

**Figure S3.** Expanded $^1$H-NMR spectrum ($\delta_H$ 5.4–3.6) of cebulactam $A_3$ (**1**) at 700 MHz in DMSO-$d_6$.

**Figure S4.** Expanded $^1$H-NMR spectrum ($\delta_H$ 2.1–0.9) of cebulactam $A_3$ (**1**) at 700 MHz in DMSO-$d_6$.

**Figure S5.** $^{13}$C-NMR spectrum of cebulactam $A_3$ (**1**) at 175 MHz in DMSO-$d_6$.

**Figure S6.** COSY spectrum of cebulactam $A_3$ (**1**) in DMSO-$d_6$.

**Figure S7.** HSQC spectrum of cebulactam $A_3$ (**1**) in DMSO-$d_6$.

**Figure S8.** HMBC spectrum of cebulactam $A_3$ (**1**) in DMSO-$d_6$.

**Figure S9.** ROESY spectrum of cebulactam $A_3$ (**1**) in DMSO-$d_6$.

**Figure S10.** TOCSY spectrum of cebulactam $A_3$ (**1**) in DMSO-$d_6$.

**Figure S11.** $^1$H-NMR spectrum of *S*-MTPA ester (**1a**) at 600 MHz in DMSO-$d_6$.

**Figure S12.** COSY spectrum of *S*-MTPA ester (**1a**) in DMSO-$d_6$.

**Figure S13.** $^1$H-NMR spectrum of *R*-MTPA ester (**1b**) at 600 MHz in DMSO-$d_6$.

**Figure S14.** COSY spectrum of *R*-MTPA ester (**1b**) in DMSO-$d_6$.

**Figure S15.** $^1$H-NMR spectrum of cebulactam $A_1$ (**2**) at 700 MHz in DMSO-$d_6$.

**Figure S16.** Expanded $^1$H-NMR spectrum ($\delta_H$ 6.9–6.4) of cebulactam $A_1$ (**2**) at 700 MHz in DMSO-$d_6$.

**Figure S17.** Expanded $^1$H-NMR spectrum ($\delta_H$ 4.9–2.9) of cebulactam $A_1$ (**2**) at 700 MHz in DMSO-$d_6$.

**Figure S18.** Expanded $^1$H-NMR spectrum ($\delta_H$ 1.9–0.6) of cebulactam $A_1$ (**2**) at 700 MHz in DMSO-$d_6$.

**Figure S19.** $^{13}$C-NMR spectrum of cebulactam $A_1$ (**2**) at 175 MHz in DMSO-$d_6$.

**Figure S20.** COSY spectrum of cebulactam $A_1$ (**2**) in DMSO-$d_6$.

**Figure S21.** HSQC spectrum of cebulactam $A_1$ (**2**) in DMSO-$d_6$.

**Figure S22.** HMBC spectrum of cebulactam $A_1$ (**2**) in DMSO-$d_6$.

**Figure S23.** ROESY spectrum of cebulactam $A_1$ (**2**) in DMSO-$d_6$.

**Figure S24.** TOCSY spectrum of cebulactam $A_1$ (**2**) in DMSO-$d_6$.

**Figure S25.** $^1$H-NMR spectrum of cebulactam $A_2$ (**3**) at 700 MHz in DMSO-$d_6$.

**Figure S26.** Expanded $^1$H-NMR spectrum ($\delta_H$ 6.7–6.5) of cebulactam $A_2$ (**3**) at 700 MHz in DMSO-$d_6$.

**Figure S27.** Expanded $^1$H-NMR spectrum ($\delta_H$ 5.0–3.2) of cebulactam $A_2$ (**3**) at 700 MHz in DMSO-$d_6$.

**Figure S28.** Expanded $^1$H-NMR spectrum ($\delta_H$ 2.0–0.9) of cebulactam $A_2$ (**3**) at 700 MHz in DMSO-$d_6$.

**Figure S29.** $^{13}$C-NMR spectrum of cebulactam $A_2$ (**3**) at 175 MHz in DMSO-$d_6$.

**Figure S30.** COSY spectrum of cebulactam $A_2$ (**3**) in DMSO-$d_6$.

**Figure S31.** HSQC spectrum of cebulactam $A_2$ (**3**) in DMSO-$d_6$.

**Figure S32.** HMBC spectrum of cebulactam $A_2$ (**3**) in DMSO-$d_6$.

**Figure S33.** ROESY spectrum of cebulactam $A_2$ (**3**) in DMSO-$d_6$.

**Figure S34.** TOCSY spectrum of cebulactam $A_2$ (**3**) in DMSO-$d_6$.

**Figure S35.** $^1$H-NMR spectrum of shengliangmycin B (**4**) at 700 MHz in DMSO-$d_6$.

**Figure S36.** Expanded $^1$H-NMR spectrum ($\delta_H$ 6.9–6.3) of shengliangmycin B (**4**) at 700 MHz in DMSO-$d_6$.

**Figure S37.** Expanded $^1$H-NMR spectrum ($\delta_H$ 5.3–2.5) of shengliangmycin B (**4**) at 700 MHz in DMSO-$d_6$.

**Figure S38.** Expanded $^1$H-NMR spectrum ($\delta_H$ 1.9–1.1) of shengliangmycin B (**4**) at 700 MHz in DMSO-$d_6$.

**Figure S39.** $^{13}$C-NMR spectrum of shengliangmycin B (**4**) at 175 MHz in DMSO-$d_6$.

**Figure S40.** COSY spectrum of shengliangmycin B (**4**) in DMSO-$d_6$.

**Figure S41.** HSQC spectrum of shengliangmycin B (**4**) in DMSO-$d_6$.

**Figure S42.** HMBC spectrum of shengliangmycin B (**4**) in DMSO-$d_6$.

**Figure S43.** ROESY spectrum of shengliangmycin B (**4**) in DMSO-$d_6$.

**Figure S44.** TOCSY spectrum of shengliangmycin B (**4**) in DMSO-$d_6$.

**Figure S45.** $^1$H-NMR spectrum of shengliangmycin D (**5**) at 700 MHz in DMSO-$d_6$.

**Figure S46.** Expanded $^1$H-NMR spectrum ($\delta_H$ 6.6–6.0) of shengliangmycin D (**5**) at 700 MHz in DMSO-$d_6$.

**Figure S47.** Expanded $^1$H-NMR spectrum ($\delta_H$ 5.4–2.2) of shengliangmycin D (**5**) at 700 MHz in DMSO-$d_6$.

**Figure S48.** Expanded $^1$H-NMR spectrum ($\delta_H$ 1.8–0.6) of shengliangmycin D (**5**) at 700 MHz in DMSO-$d_6$.

**Figure S49.** $^{13}$C-NMR spectrum of shengliangmycin D (**5**) at 175 MHz in DMSO-$d_6$.

**Figure S50.** COSY spectrum of shengliangmycin D (**5**) in DMSO-$d_6$.

**Figure S51.** HSQC spectrum of shengliangmycin D (**5**) in DMSO-$d_6$.

**Figure S52.** HMBC spectrum of shengliangmycin D (**5**) in DMSO-$d_6$.

**Figure S53.** ROESY spectrum of shengliangmycin D (**5**) in DMSO-$d_6$.

**Figure S54.** TOCSY spectrum of shengliangmycin D (**5**) in DMSO-$d_6$.

**Figure S55.** UV spectrum of cebulactam $A_3$ (**1**).

**Figure S56.** HR-ESI-MS data of cebulactam $A_3$ (**1**).

**Figure S57.** FT-IR spectrum of cebulactam $A_3$ (**1**).

**Figure S58.** The circular dichroism (CD) spectra of cebulactam $A_3$ and $A_2$ (**1** and **3**) at a concentration of 0.1 mg/mL in MeOH.

**Figure S59.** 16S rRNA gene sequence data of PG10.

**Figure S60.** Whole-genome-based phylogenetic tree of PG10.

**Table S1**. $^1$H and $^{13}$C NMR spectroscopic data of **2**–**5** in DMSO-$d_6$.

**Table S2.** Isolated strains from diverse media.

**Table S3.** Predicted functions of CDSs in the cebulactam gene cluster based on whole-genome sequencing data.

**Table S4.** Growth-inhibitory activities ($IC_{50}$) of compounds **1**–**5** against the tested fungal and bacterial strains.

**Table S5.** Isolation and cultivation media.

**Supplementary note**.

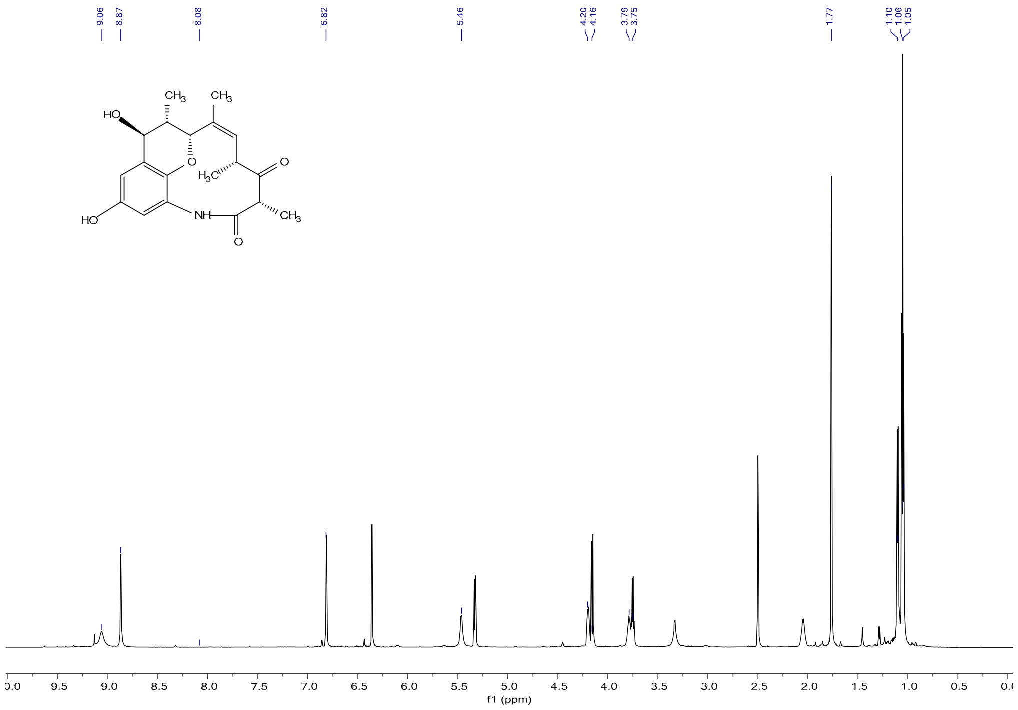


**Figure S1.** $^1$H-NMR spectrum of cebulactam A$_3$ (**1**) at 700 MHz in DMSO-*d*$_6$.

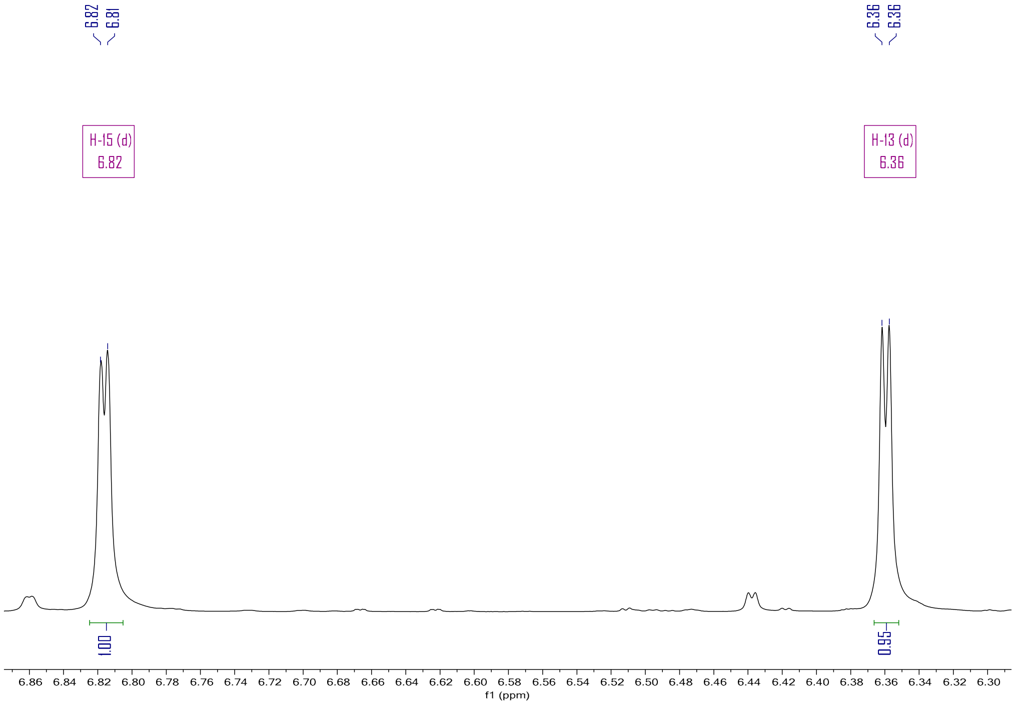


**Figure S2.** Expanded $^1$H-NMR spectrum ($\delta_H$ 6.9–6.3) of cebulactam A$_3$ (**1**) at 700 MHz in DMSO-*d*$_6$.

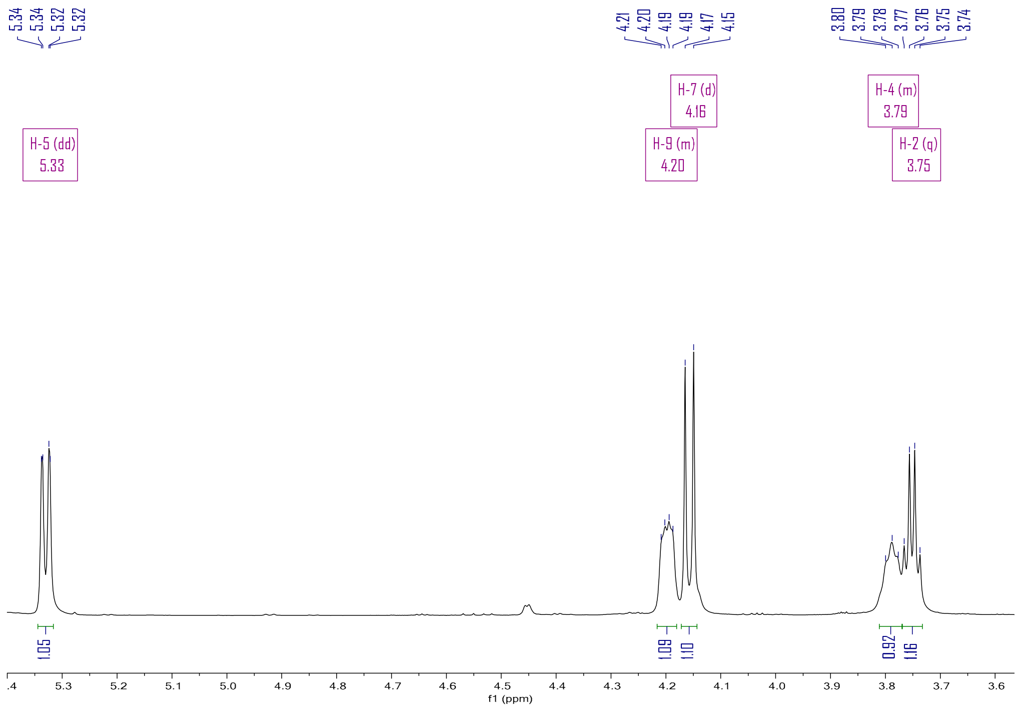


**Figure S3.** Expanded $^1$H-NMR spectrum ($\delta_H$ 5.4–3.6) of cebulactam A$_3$ (**1**) at 700 MHz in DMSO-$d_6$.

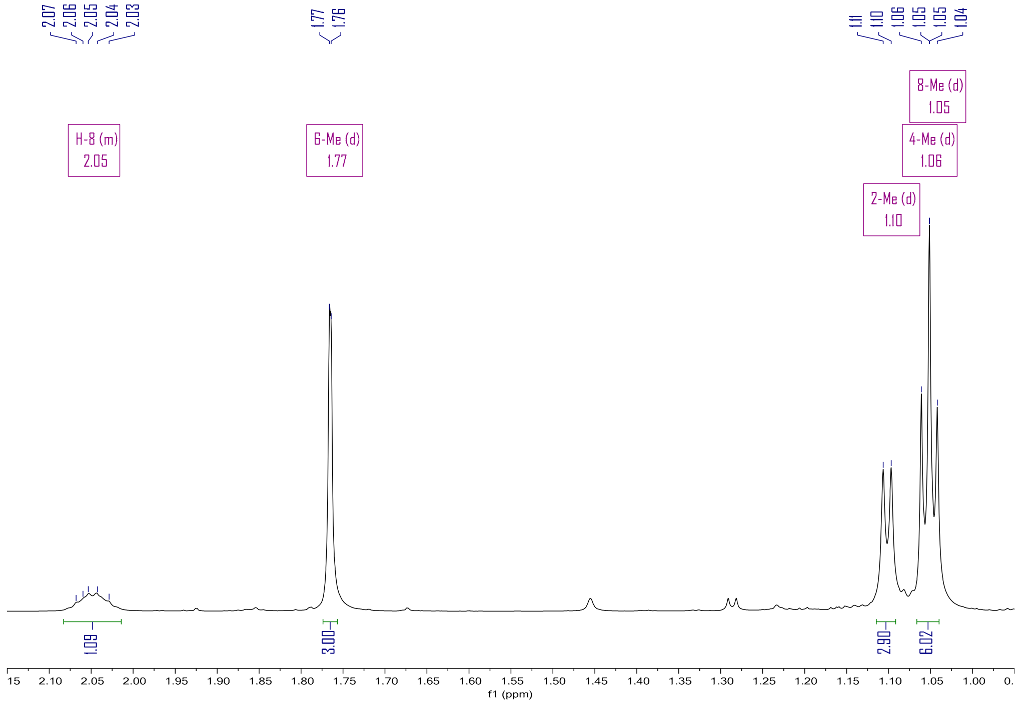


**Figure S4.** Expanded $^1$H-NMR spectrum ($\delta_H$ 2.1–0.9) of cebulactam A$_3$ (**1**) at 700 MHz in DMSO-$d_6$.

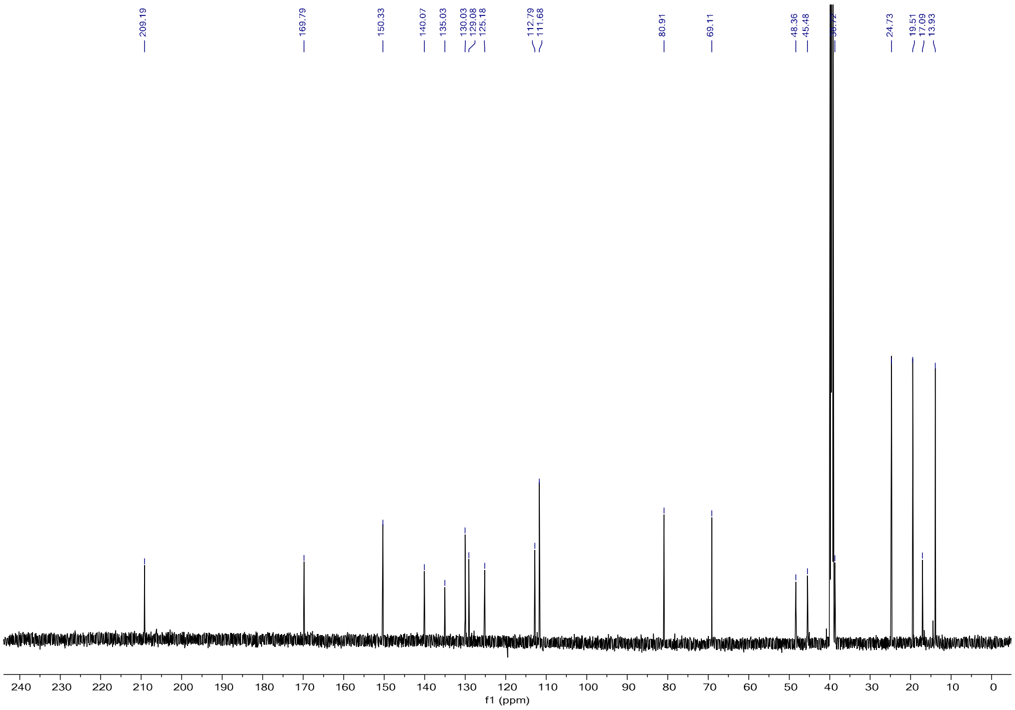


**Figure S5.** $^{13}$C-NMR spectrum of cebulactam A$_3$ (**1**) at 175 MHz in DMSO-$d_6$.

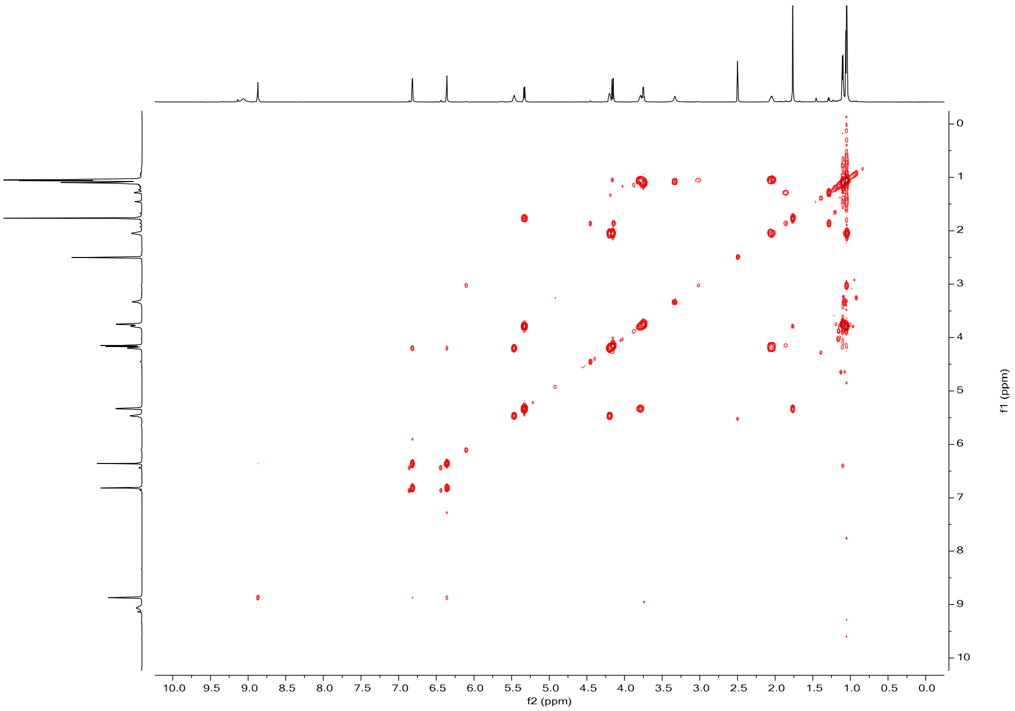


**Figure S6.** COSY spectrum of cebulactam A$_3$ (**1**) in DMSO-$d_6$.

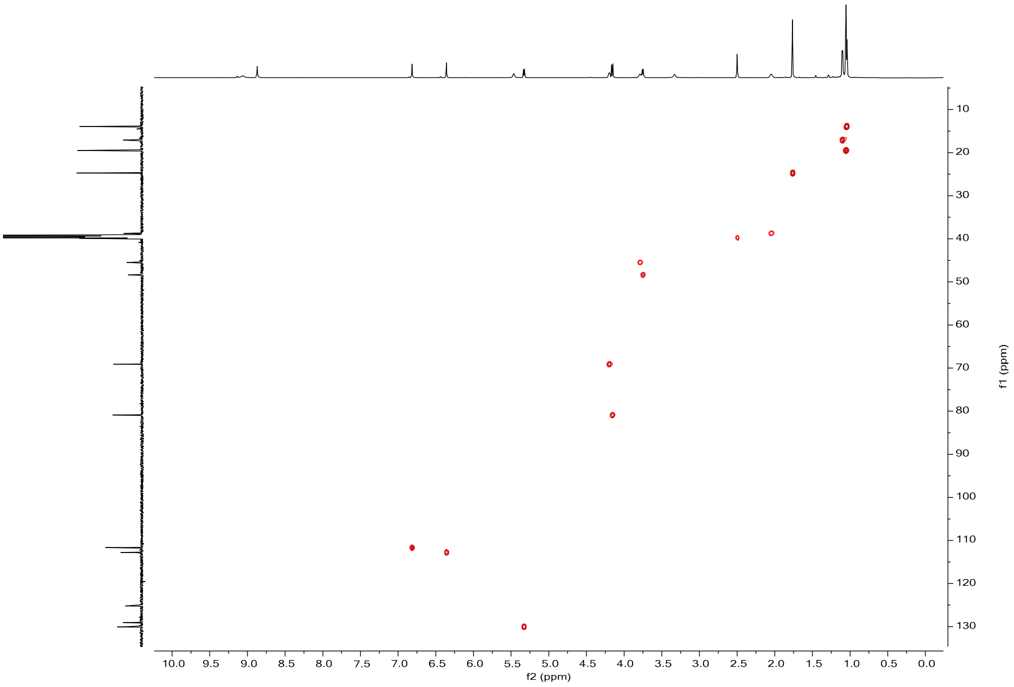


**Figure S7.** HSQC spectrum of cebulactam A3 (**1**) in DMSO-*d*6.

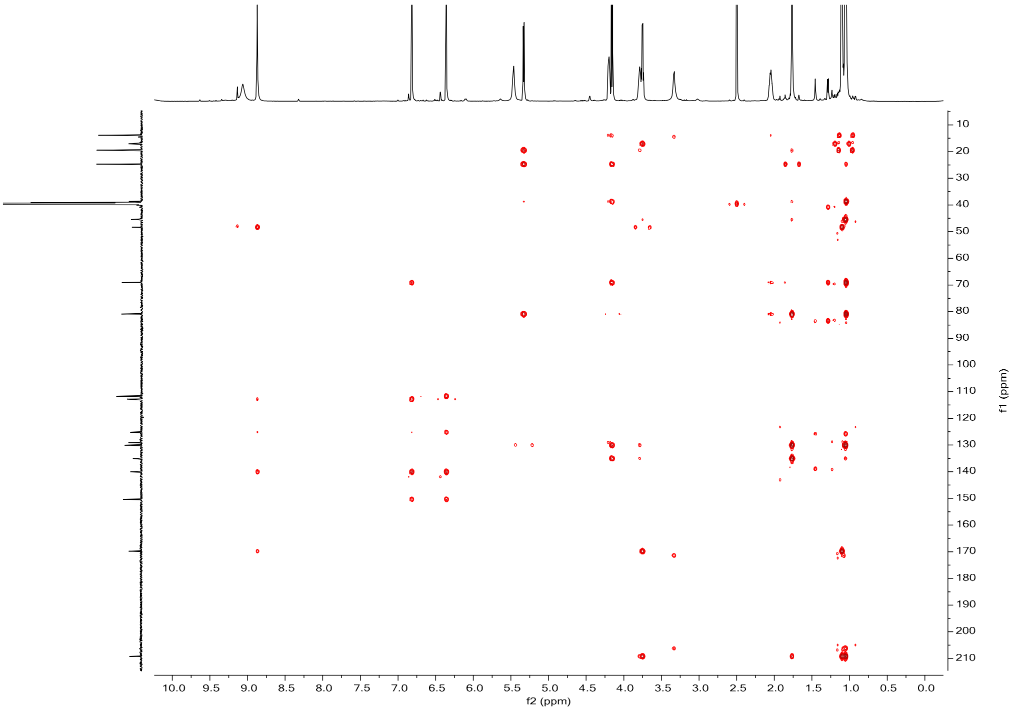


**Figure S8.** HMBC spectrum of cebulactam A3 (**1**) in DMSO-*d*6.

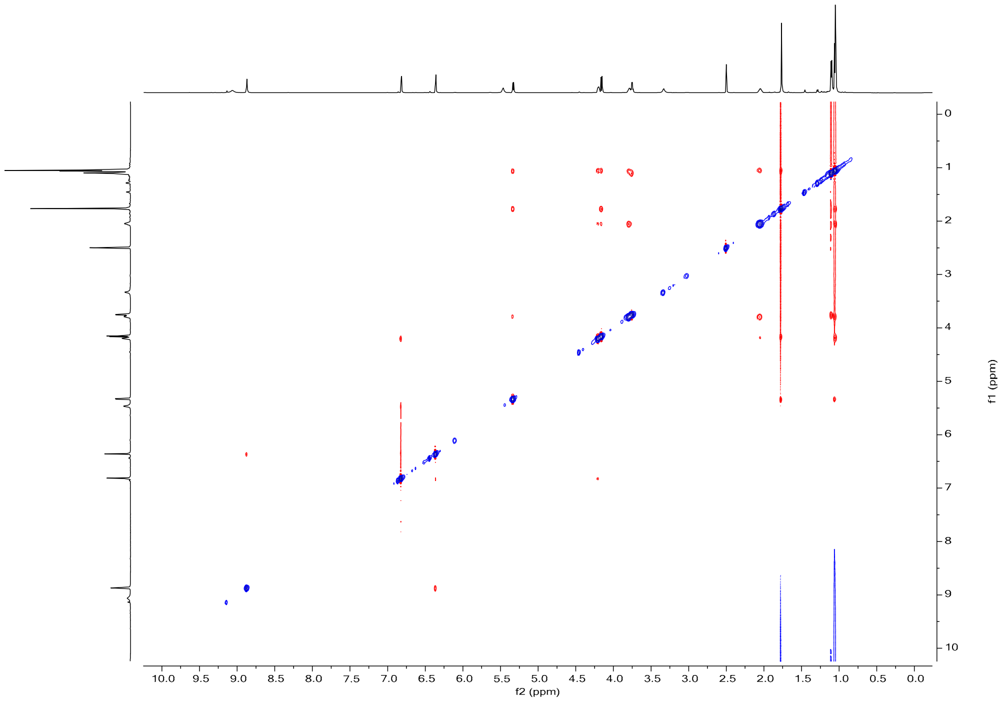


**Figure S9.** ROESY spectrum of cebulactam A3 (**1**) in DMSO-*d*6.

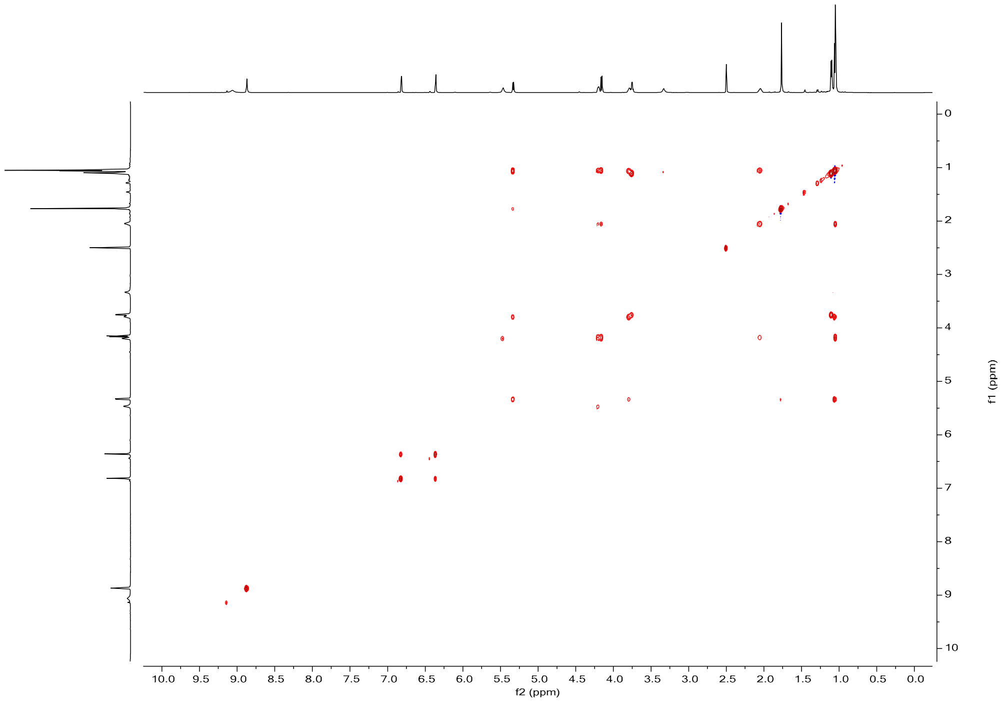


**Figure S10.** TOCSY spectrum of cebulactam A3 (**1**) in DMSO-*d*6.

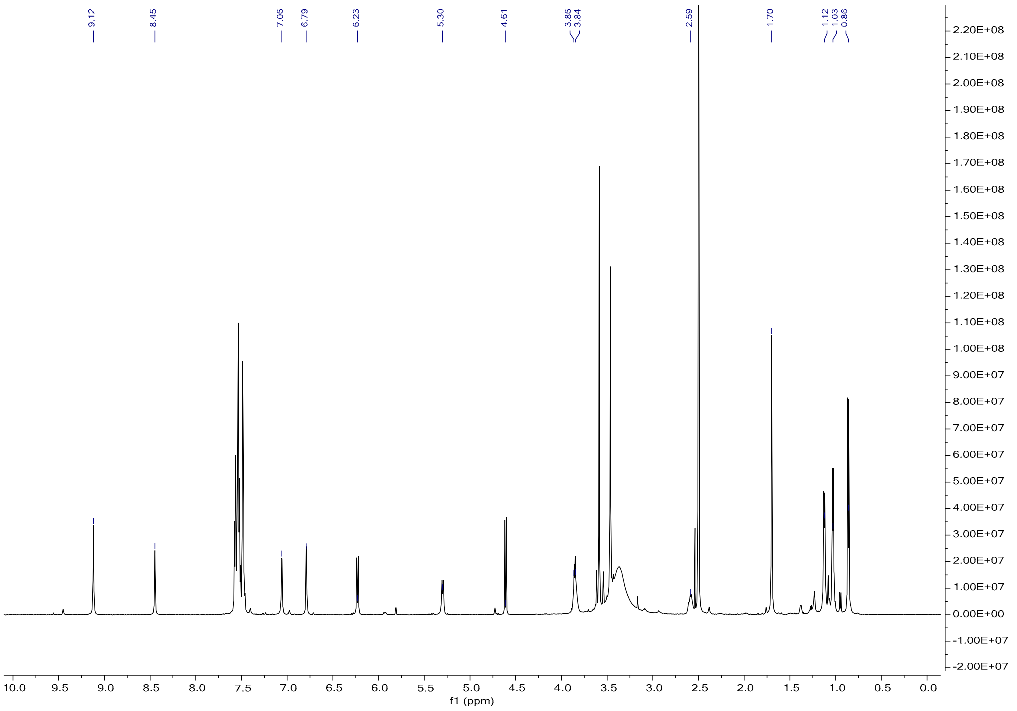


**Figure S11.** $^{1}$H-NMR spectrum of *S*-MTPA ester (**1a**) at 600 MHz in DMSO-*d*$_6$.

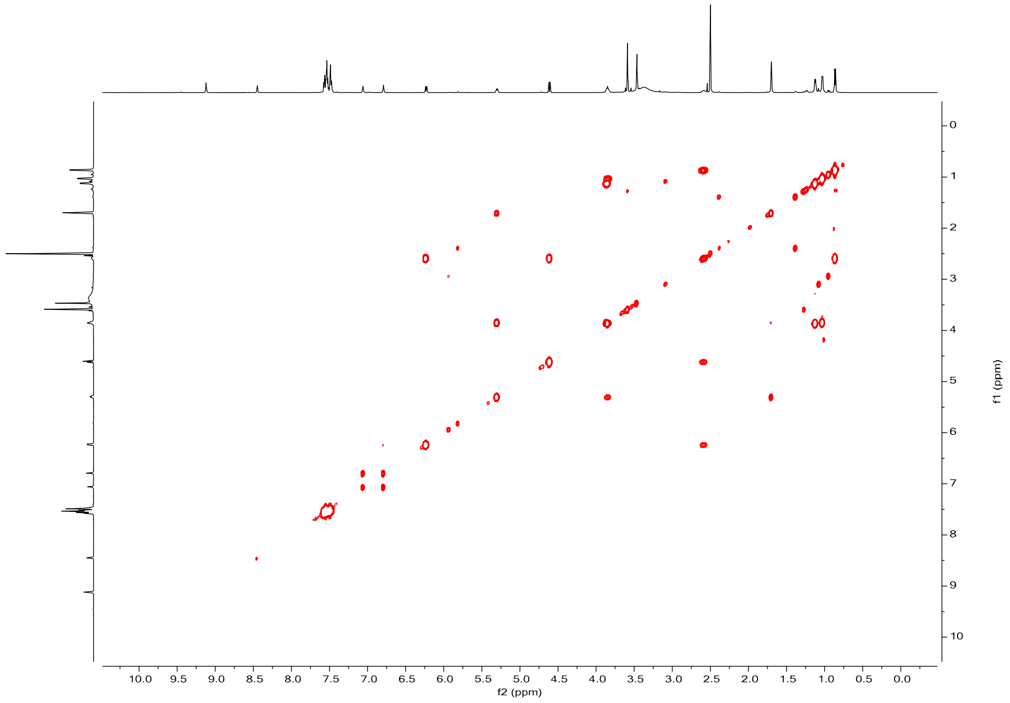


**Figure S12.** COSY spectrum of *S*-MTPA ester (**1a**) in DMSO-*d*$_6$.

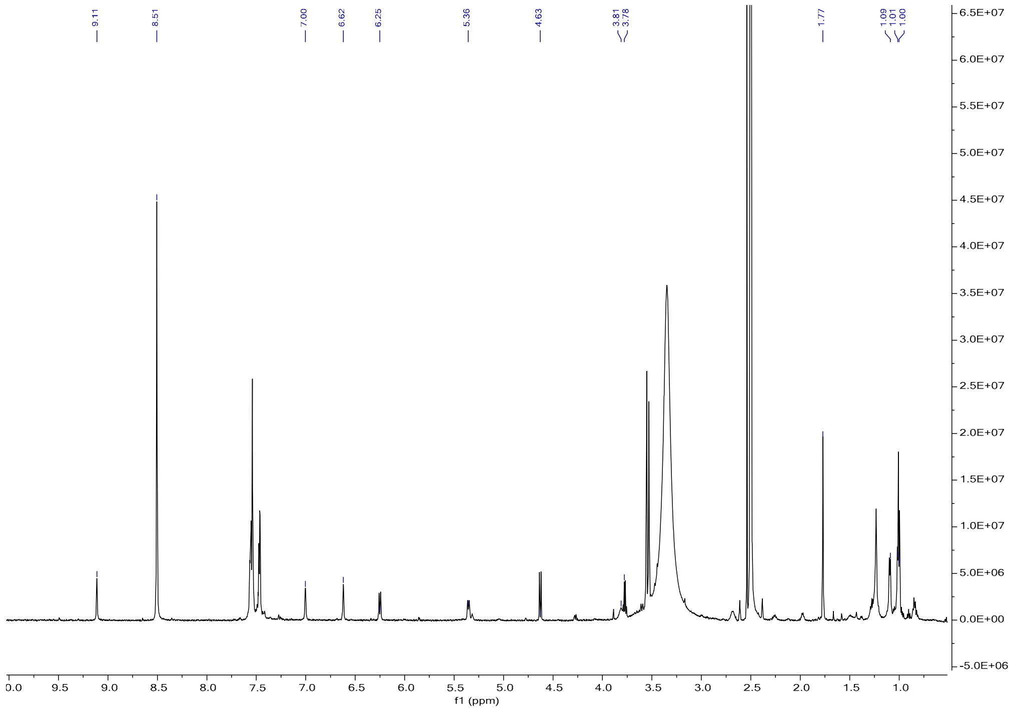


**Figure S13.** $^{1}$H-NMR spectrum of *R*-MTPA ester (**1b**) at 600 MHz in DMSO-$d_6$.

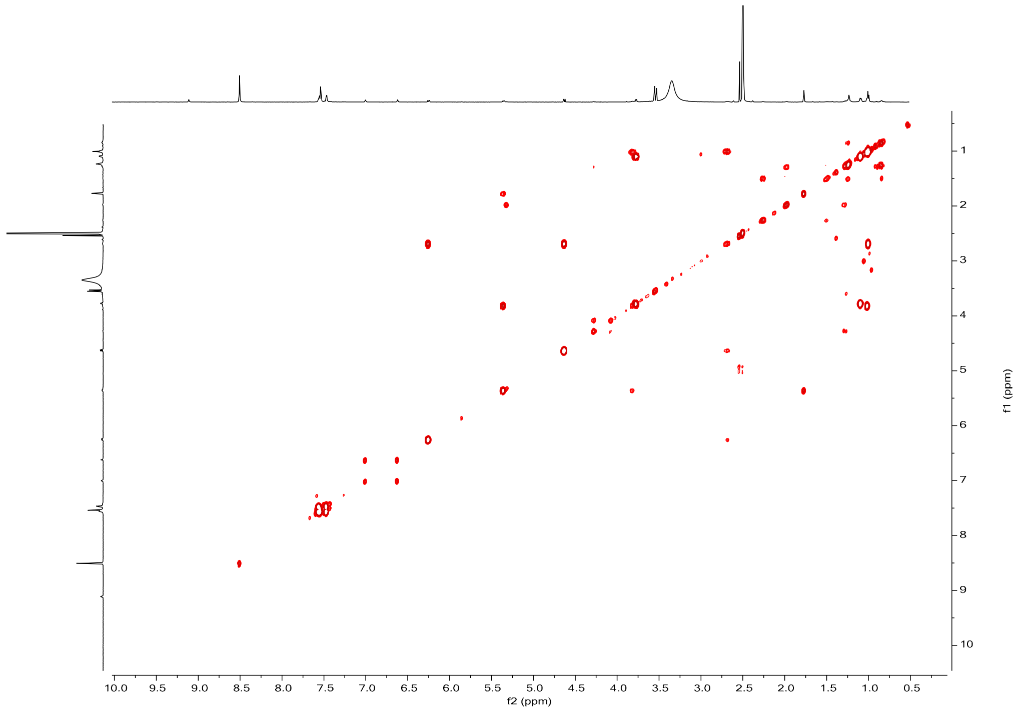


**Figure S14.** COSY spectrum of *R*-MTPA ester (**1b**) in DMSO-$d_6$.

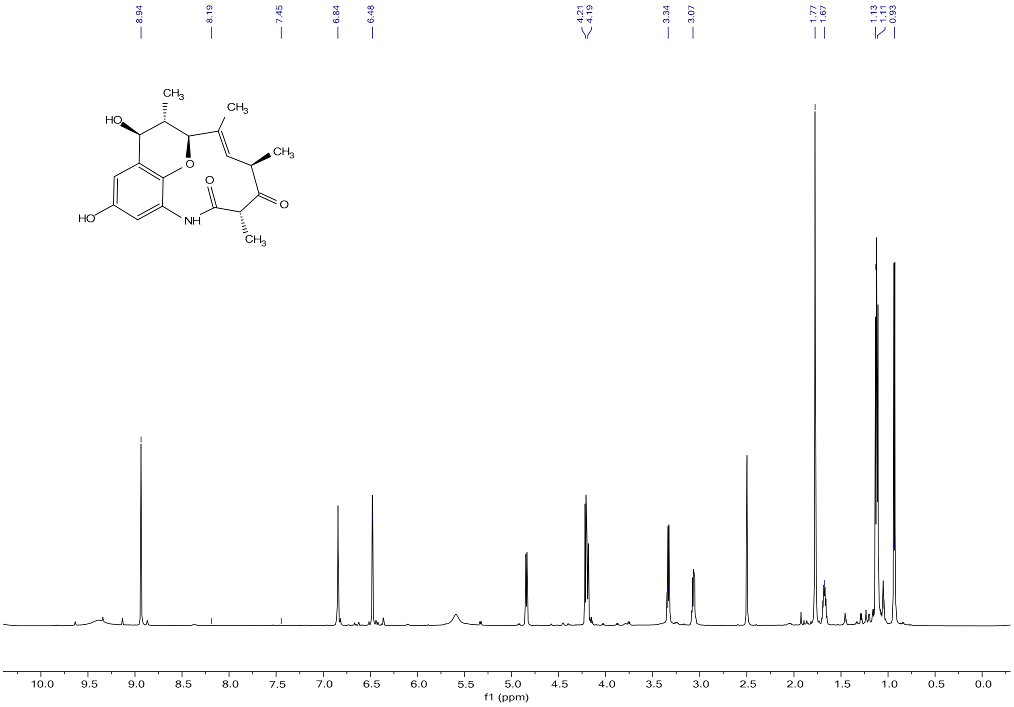


**Figure S15.** $^1$H-NMR spectrum of cebulactam A$_1$ (**2**) at 700 MHz in DMSO-$d_6$.

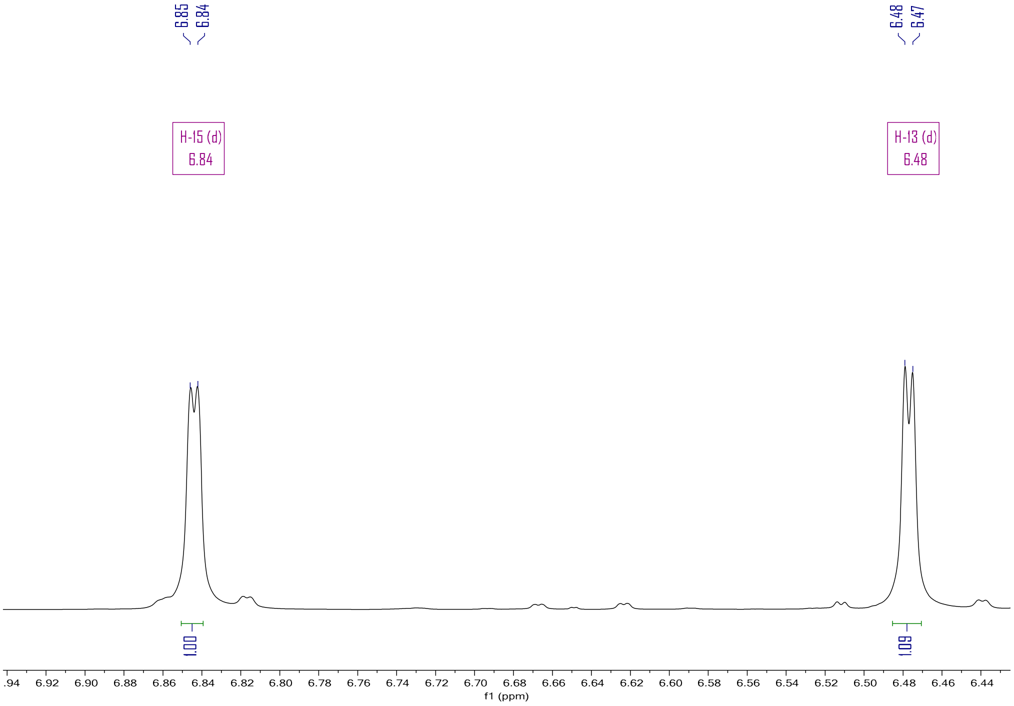


**Figure S16.** Expanded $^1$H-NMR spectrum ($\delta_H$ 6.9–6.4) of cebulactam A$_1$ (**2**) at 700 MHz in DMSO-$d_6$.

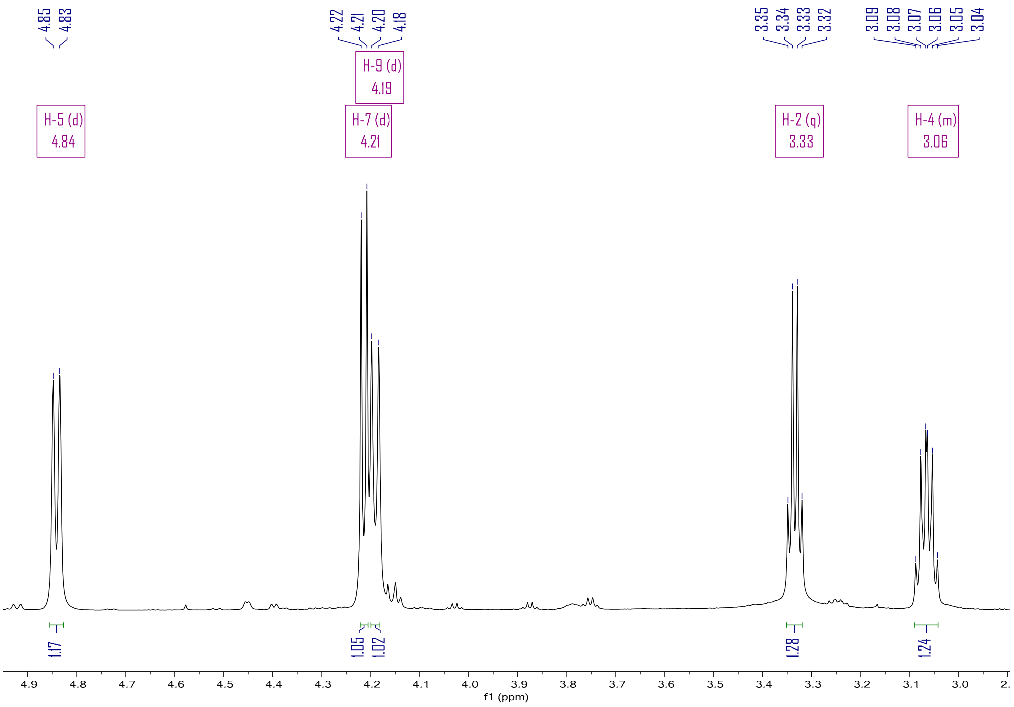


**Figure S17.** Expanded $^1$H-NMR spectrum ($\delta_H$ 4.9–2.9) of cebulactam $A_1$ (**2**) at 700 MHz in DMSO-$d_6$.

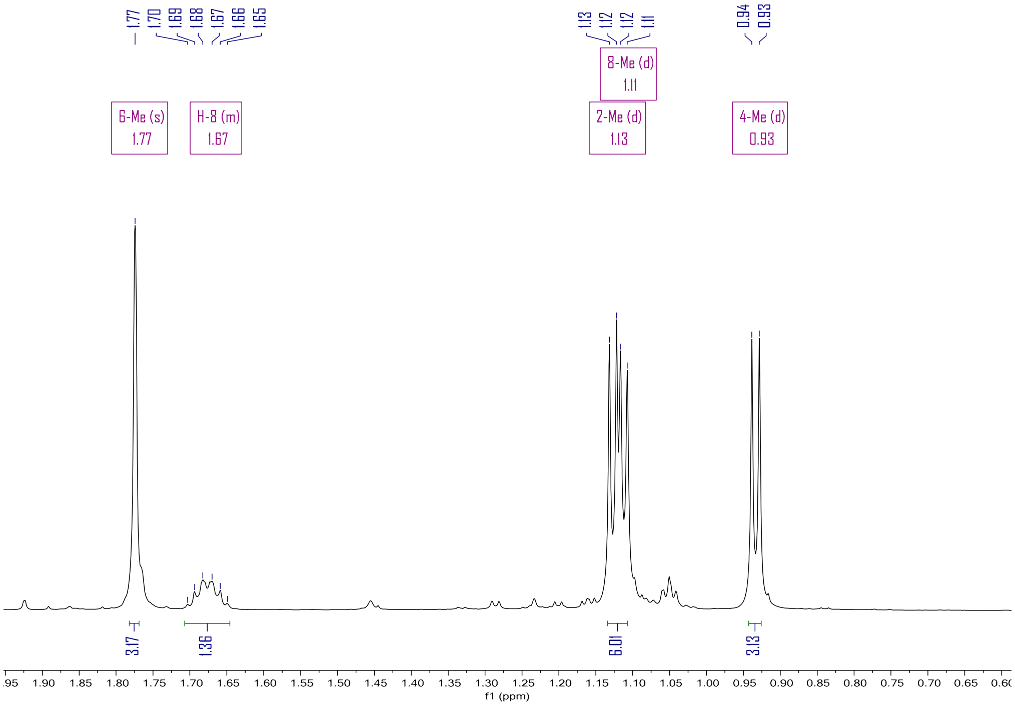


**Figure S18.** Expanded $^1$H-NMR spectrum ($\delta_H$ 1.9–0.6) of cebulactam $A_1$ (**2**) at 700 MHz in DMSO-$d_6$.

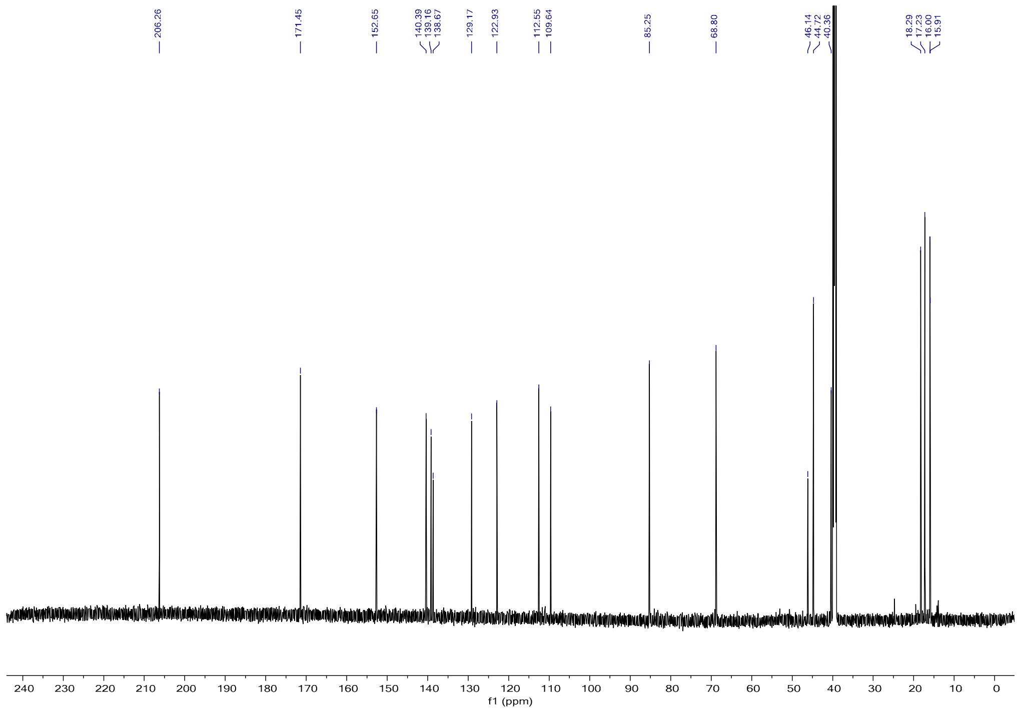


**Figure S19.** $^{13}$C-NMR spectrum of cebulactam $A_1$ (**2**) at 175 MHz in DMSO-$d_6$.

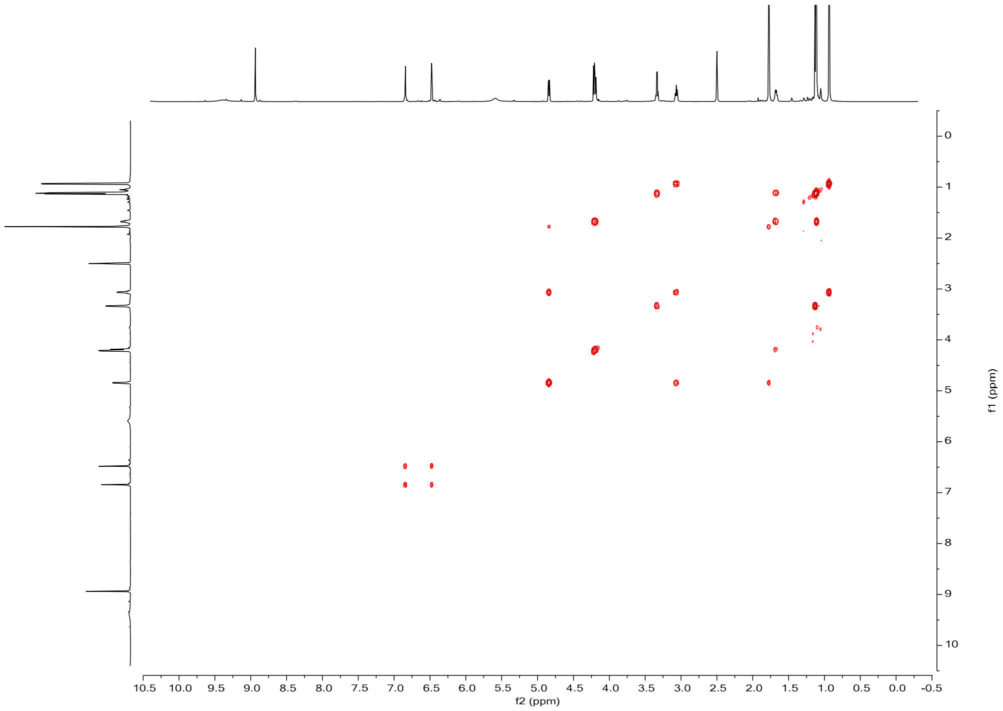


**Figure S20.** COSY spectrum of cebulactam $A_1$ (**2**) in DMSO-$d_6$.

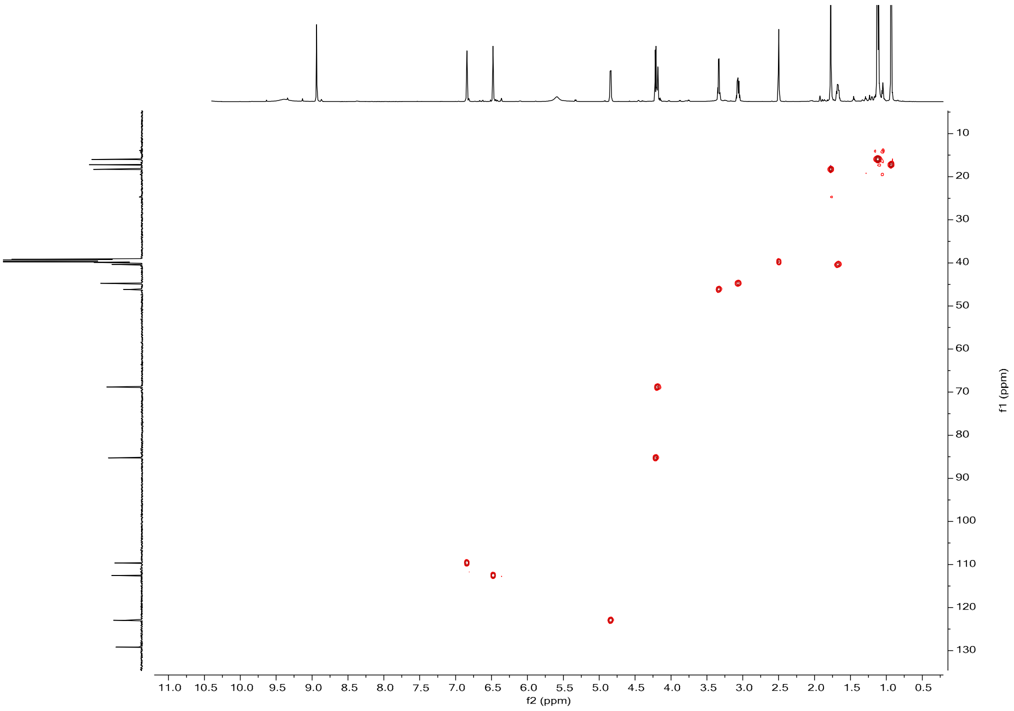


**Figure S21.** HSQC spectrum of cebulactam A1 (**2**) in DMSO-*d*6.

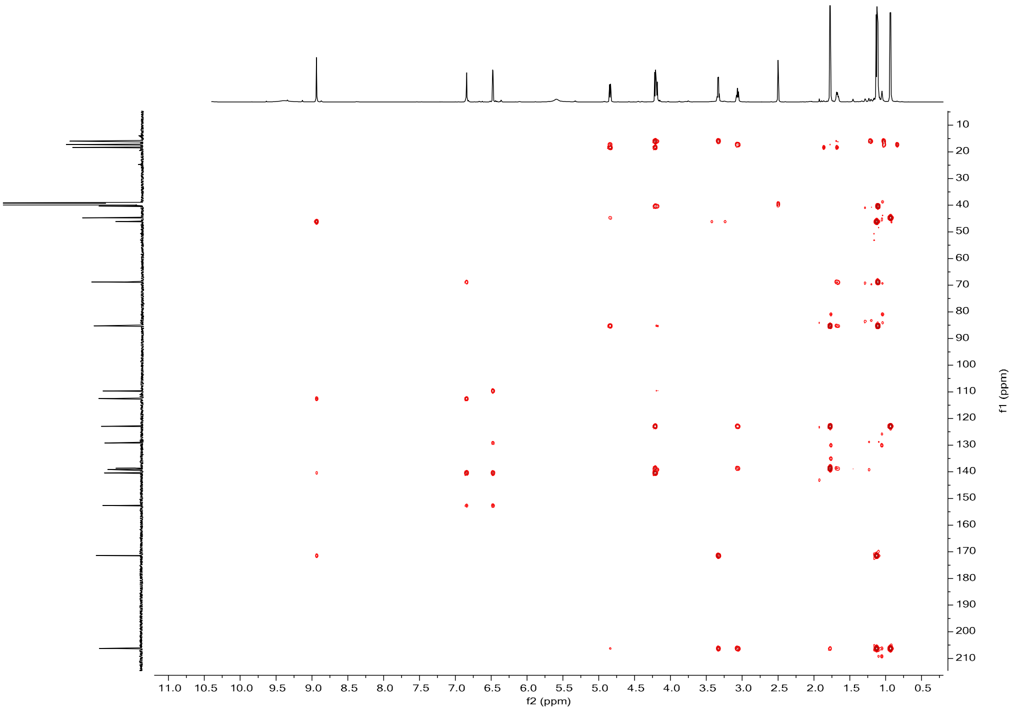


**Figure S22.** HMBC spectrum of cebulactam A1 (**2**) in DMSO-*d*6.

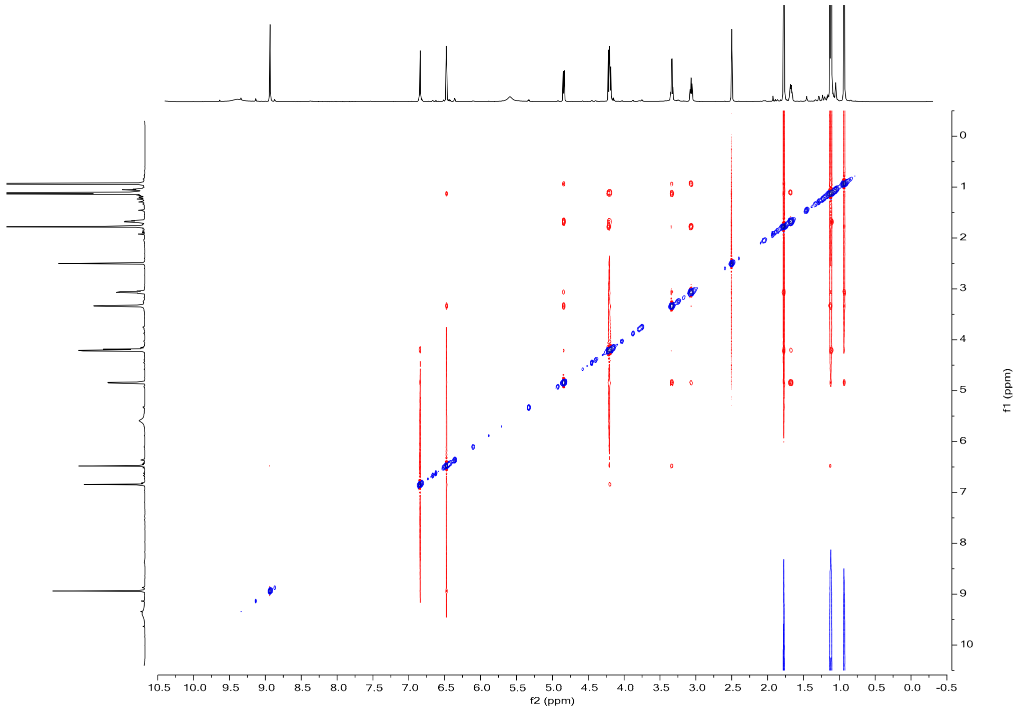


**Figure S23.** ROESY spectrum of cebulactam A1 (**2**) in DMSO-*d*6.

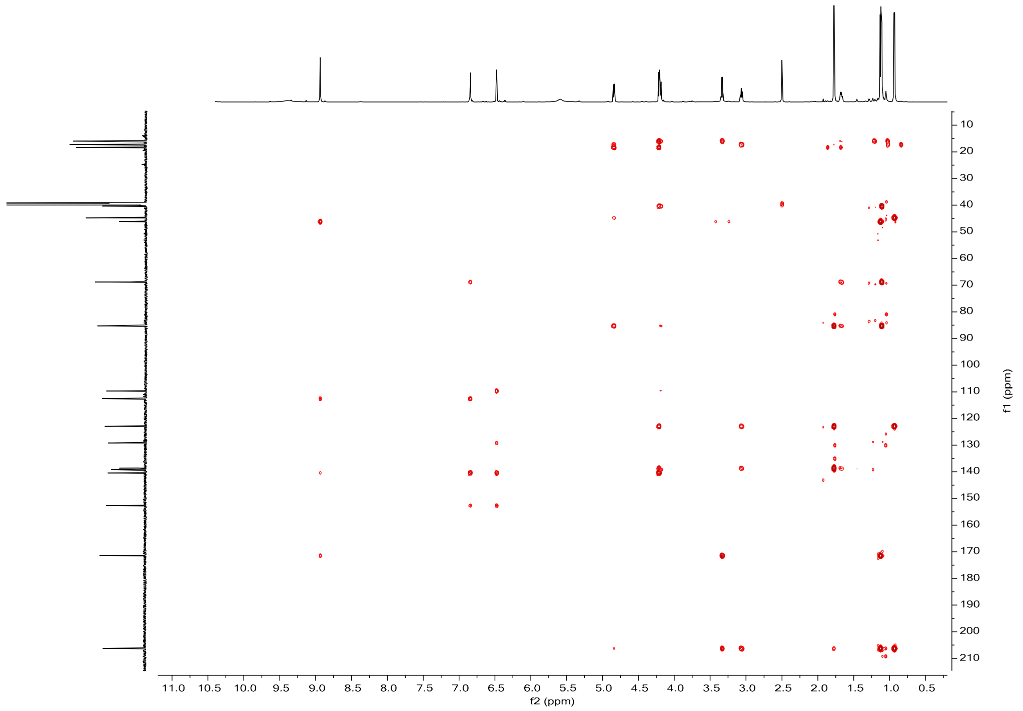


**Figure S24.** TOCSY spectrum of cebulactam A1 (**2**) in DMSO-*d*6.

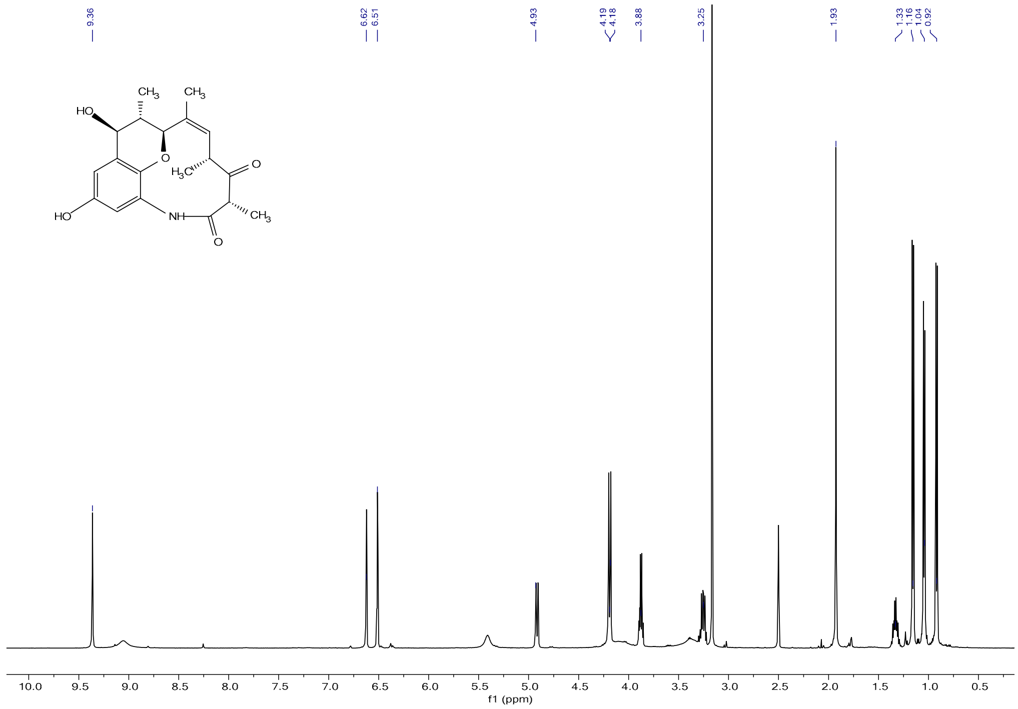

**Figure S25.** $^1$H-NMR spectrum of cebulactam A$_2$ (**3**) at 700 MHz in DMSO-$d_6$.

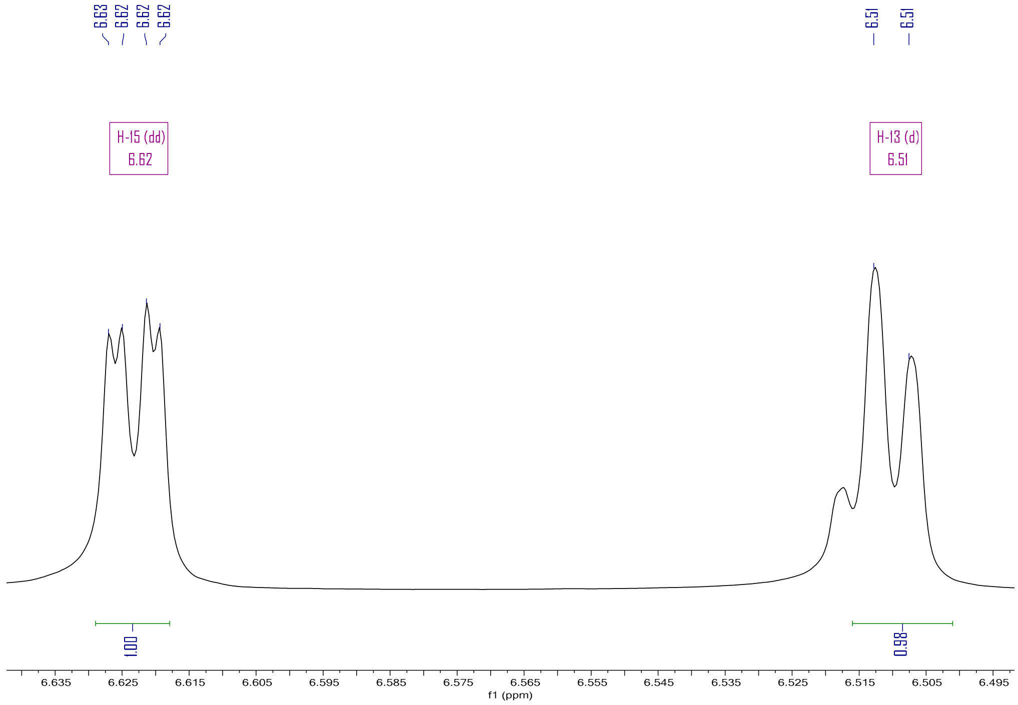


**Figure S26.** Expanded $^1$H-NMR spectrum ($\delta_H$ 6.7–6.5) of cebulactam A$_2$ (**3**) at 700 MHz in DMSO-$d_6$.

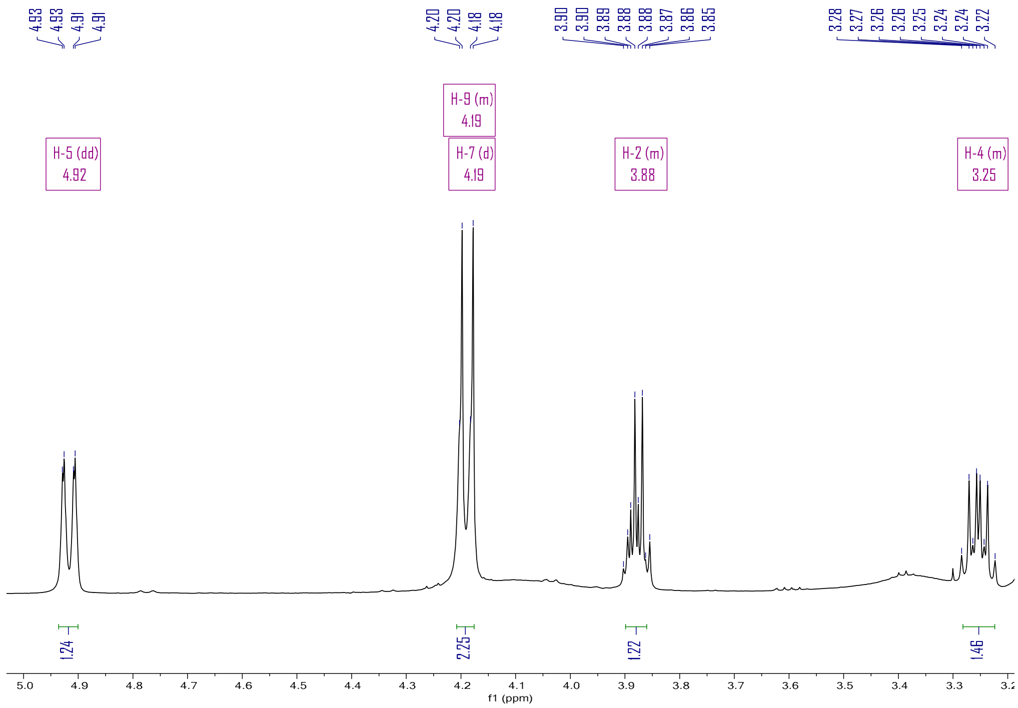


**Figure S27.** Expanded $^{1}$H-NMR spectrum ($\delta_H$ 5.0–3.2) of cebulactam $A_2$ (**3**) at 700 MHz in DMSO-$d_6$.

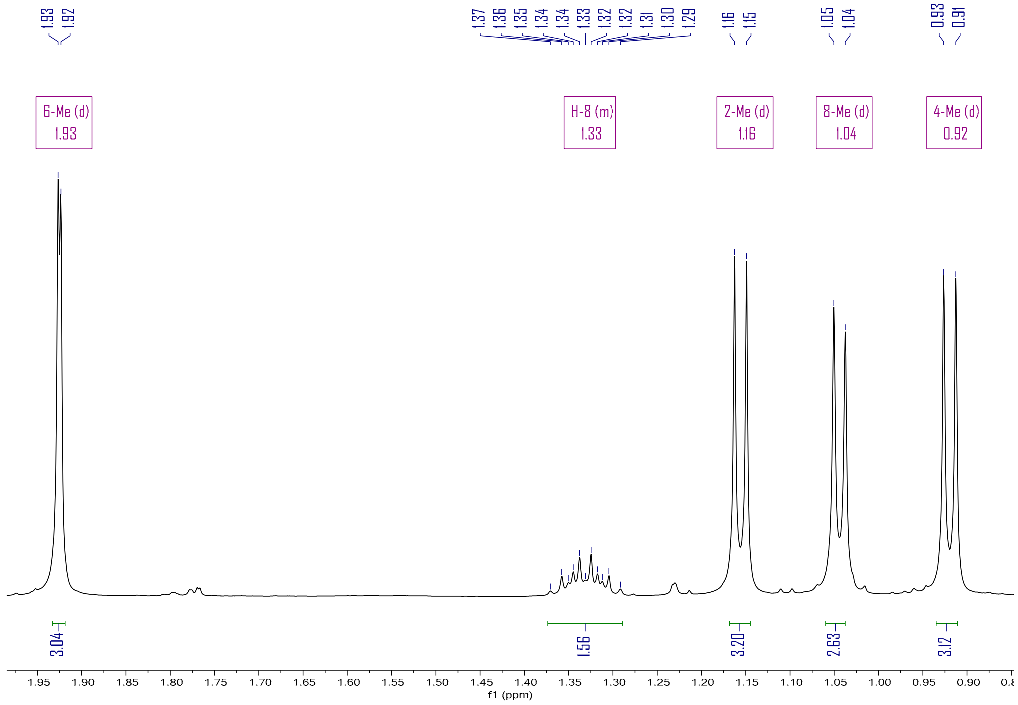


**Figure S28.** Expanded $^{1}$H-NMR spectrum ($\delta_H$ 2.0–0.9) of cebulactam $A_2$ (**3**) at 700 MHz in DMSO-$d_6$.

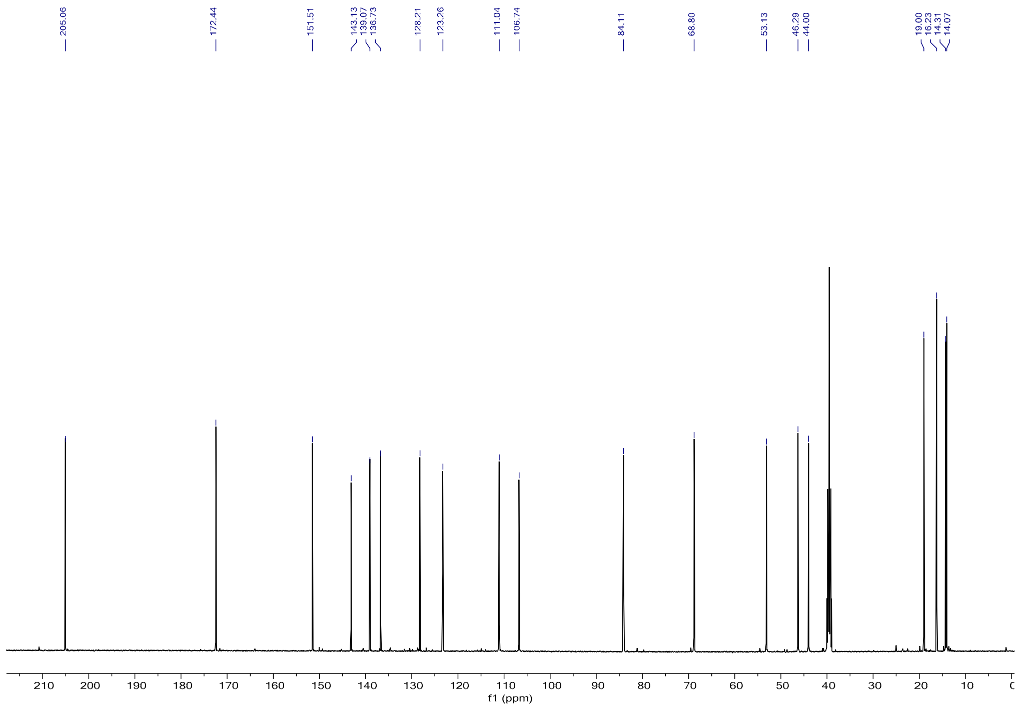


**Figure S29.** $^{13}$C-NMR spectrum of cebulactam A2 (**3**) at 175 MHz in DMSO-*d*6.

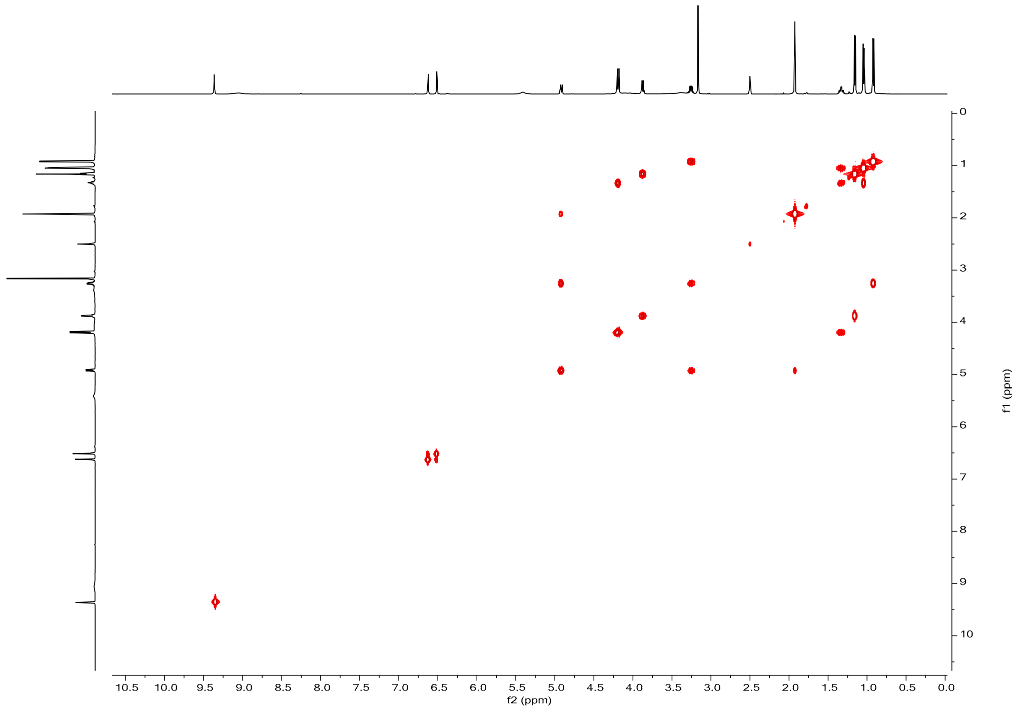


**Figure S30.** COSY spectrum of cebulactam A2 (**3**) in DMSO-*d*6.

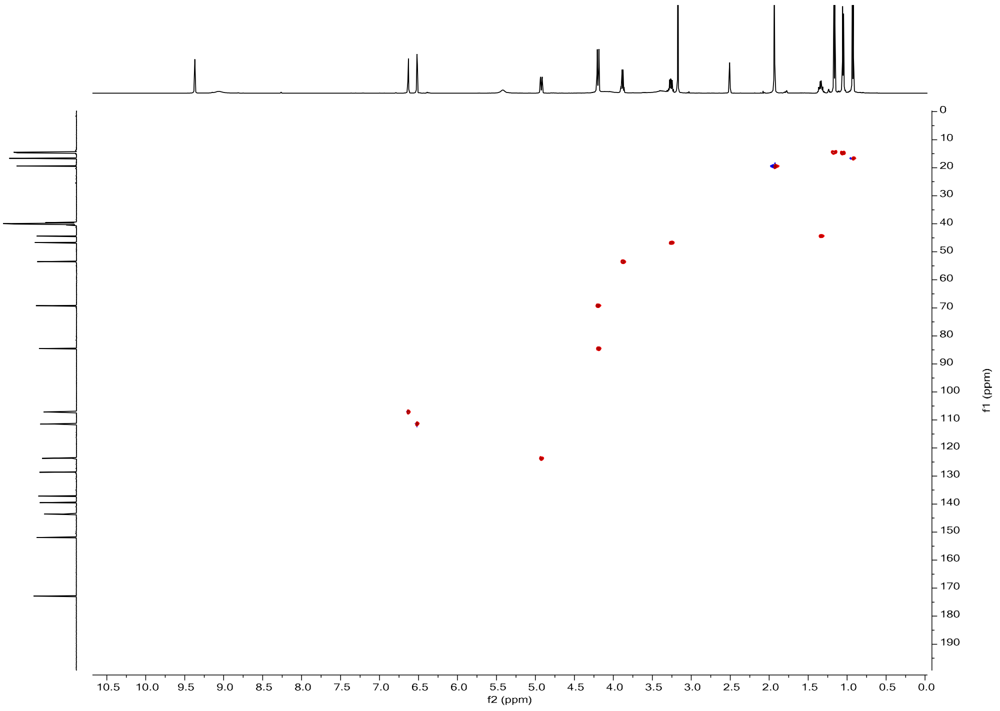


**Figure S31.** HSQC spectrum of cebulactam A2 (**3**) in DMSO-*d*6.

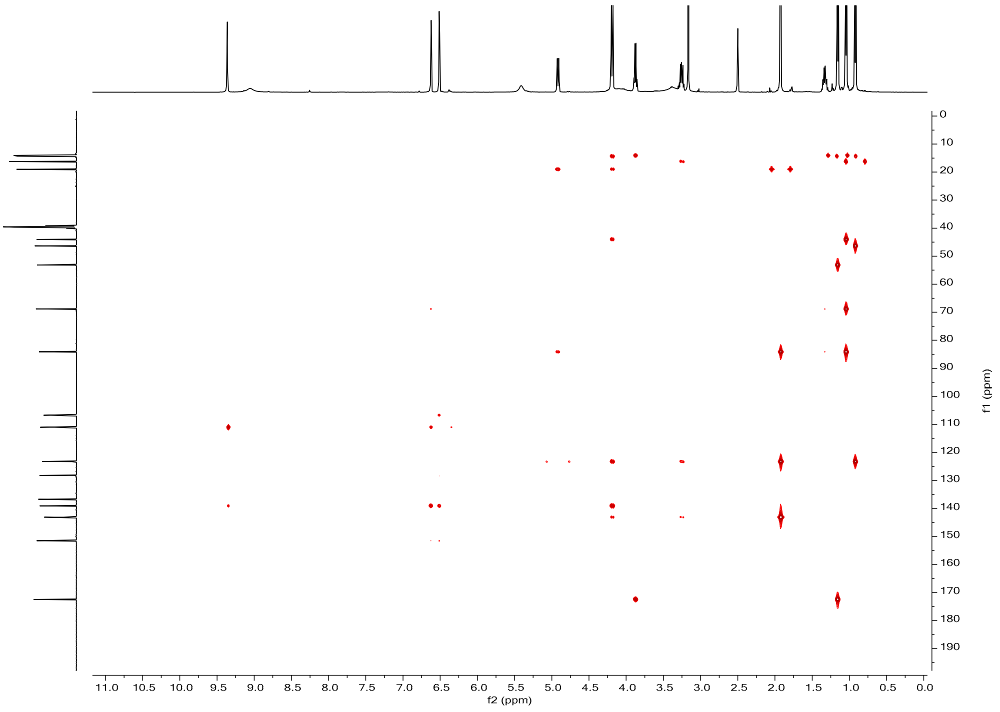


**Figure S32.** HMBC spectrum of cebulactam A2 (**3**) in DMSO-*d*6.

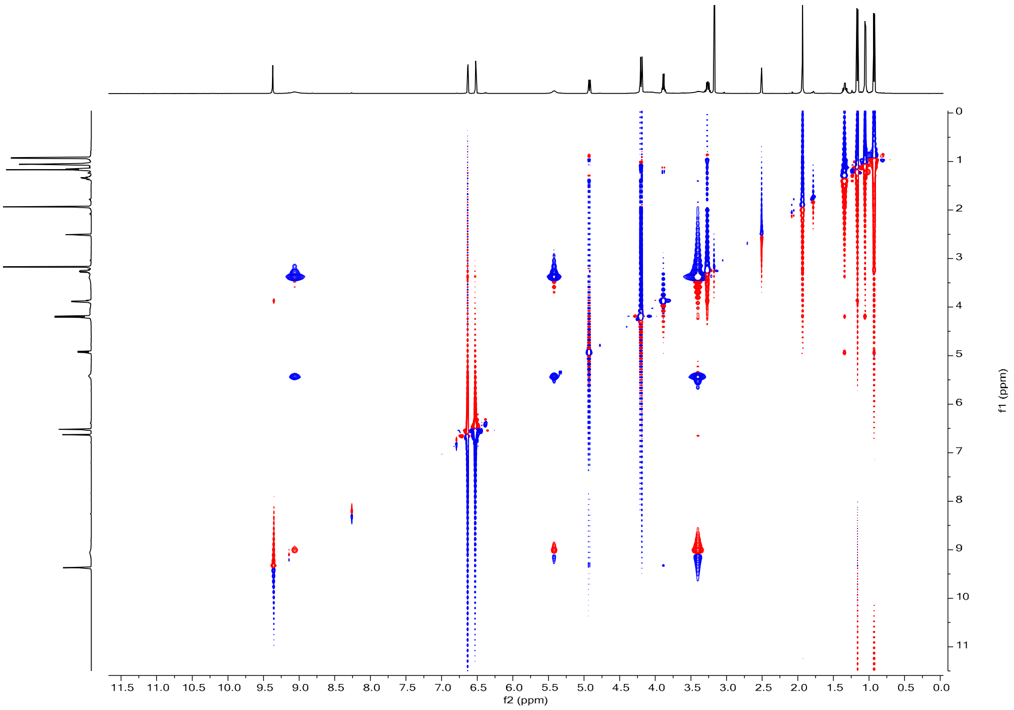


**Figure S33.** ROESY spectrum of cebulactam A2 (**3**) in DMSO-*d*6.

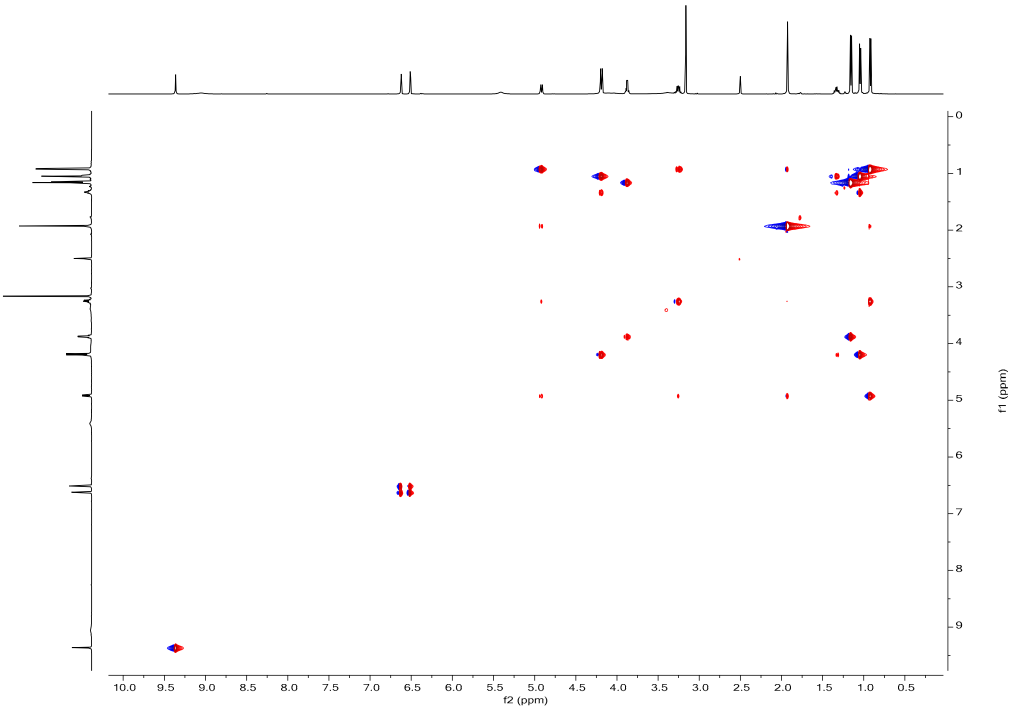


**Figure S34.** TOCSY spectrum of cebulactam A2 (**3**) in DMSO-*d*6.

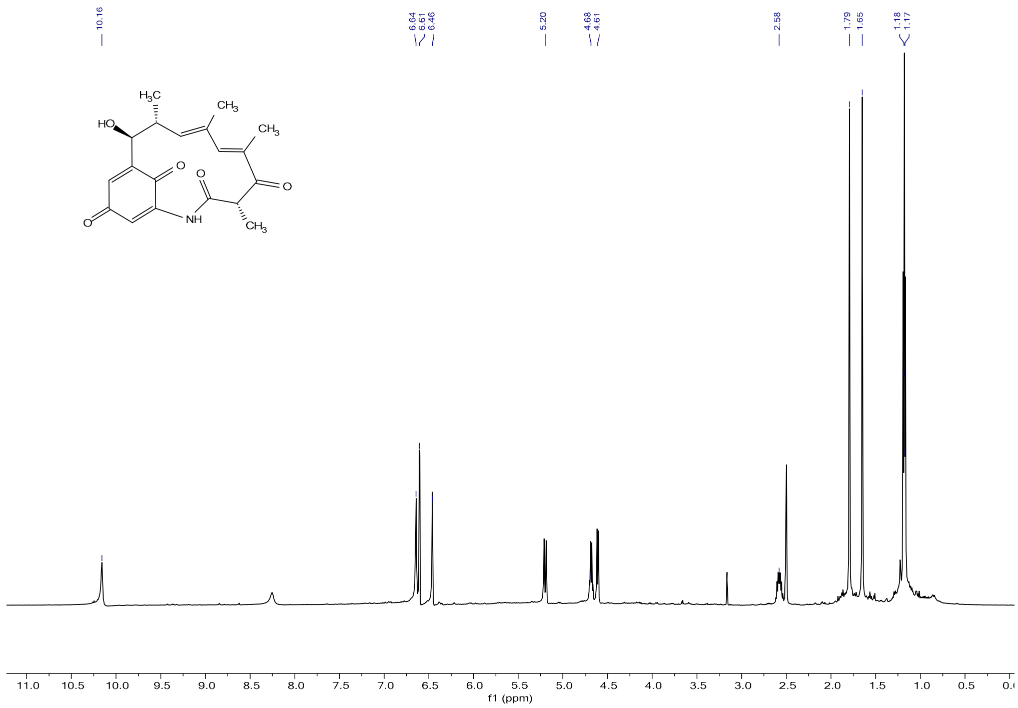


**Figure S35.** $^{1}$H-NMR spectrum of shengliangmycin B (**4**) at 700 MHz in DMSO-$d_6$.

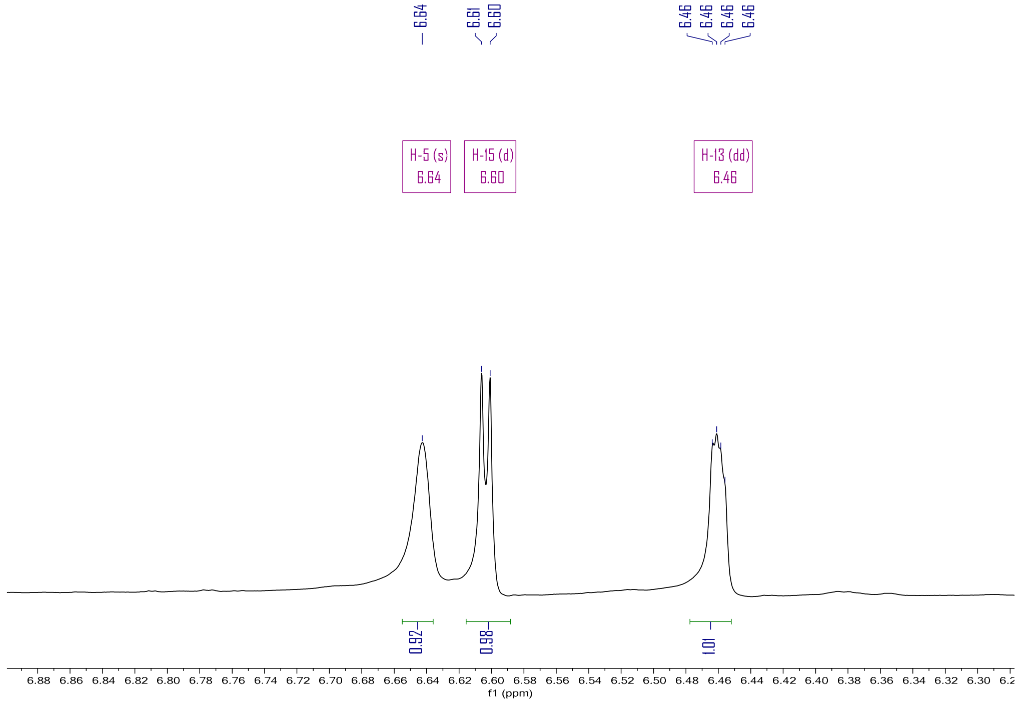


**Figure S36.** Expanded $^{1}$H-NMR spectrum ($\delta_H$ 6.9–6.3) of shengliangmycin B (**4**) at 700 MHz in DMSO-$d_6$.

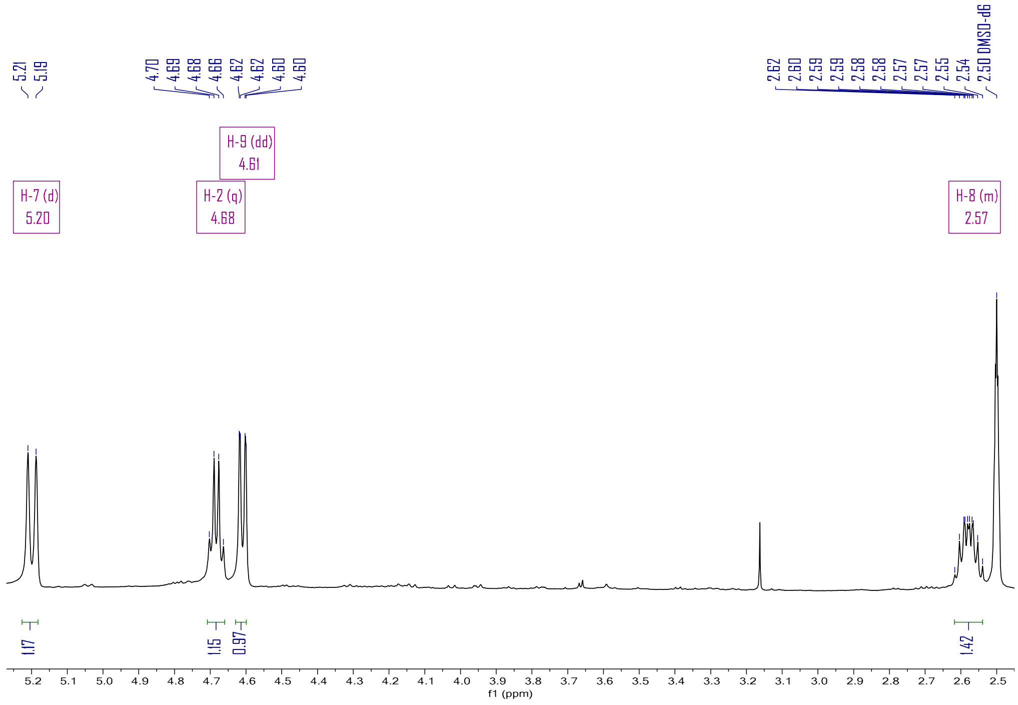


**Figure S37.** Expanded $^{1}$H-NMR spectrum ($\delta_H$ 5.3–2.5) of shengliangmycin B (**4**) at 700 MHz in DMSO-$d_6$.

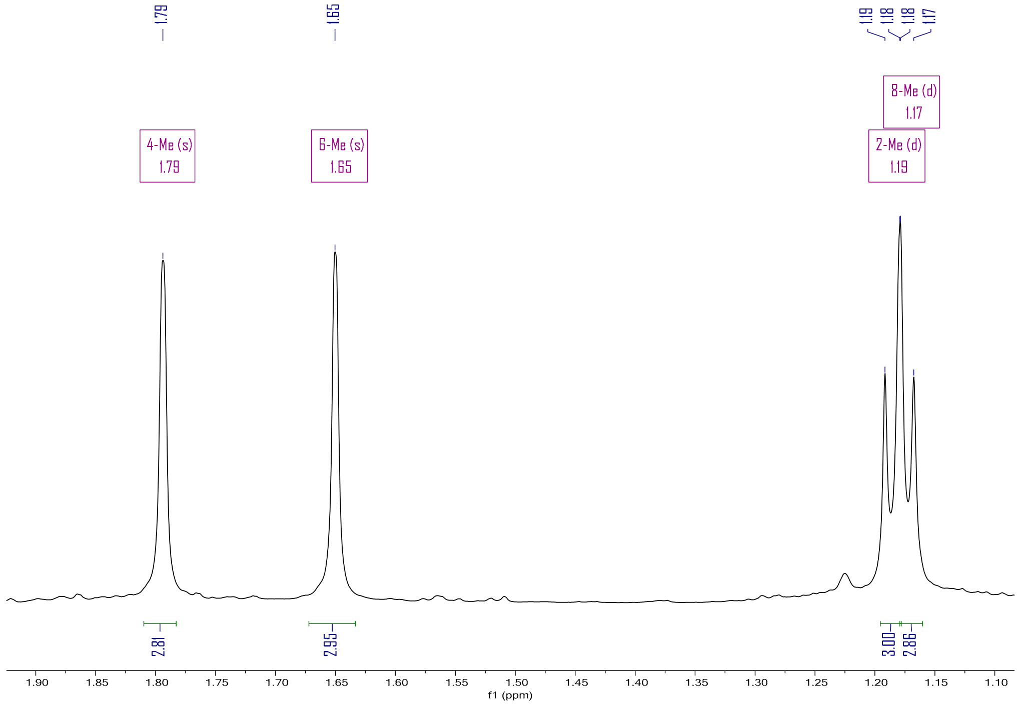


**Figure S38.** Expanded $^{1}$H-NMR spectrum ($\delta_H$ 1.9–1.1) of shengliangmycin B (**4**) at 700 MHz in DMSO-$d_6$.

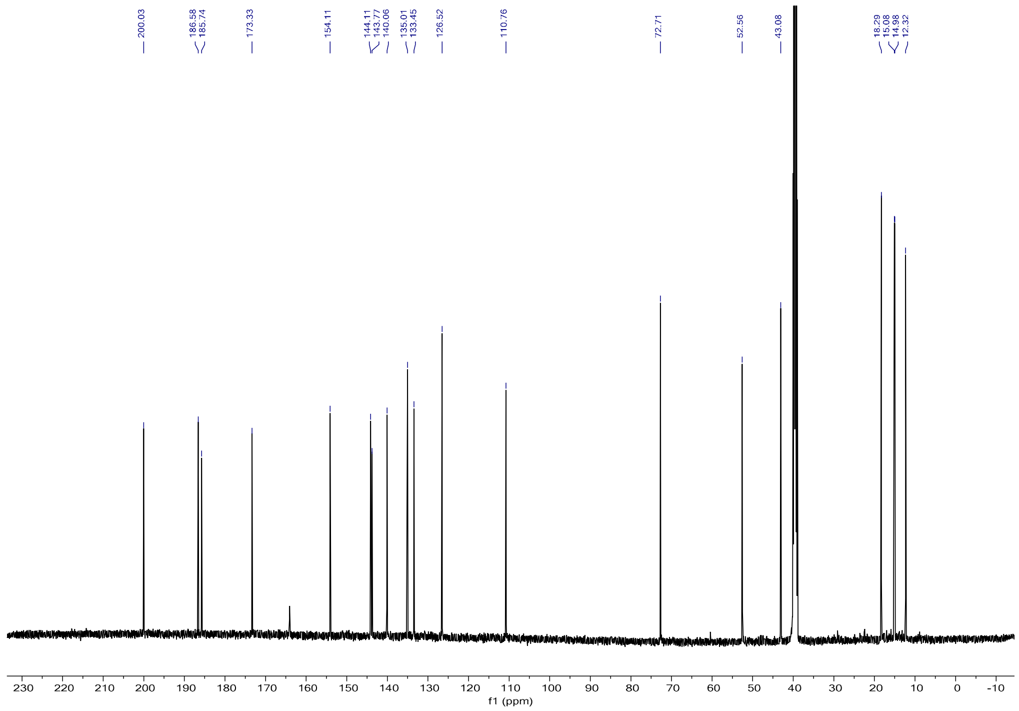


**Figure S39.** $^{13}$C-NMR spectrum of shengliangmycin B (**4**) at 175 MHz in DMSO-$d_6$.

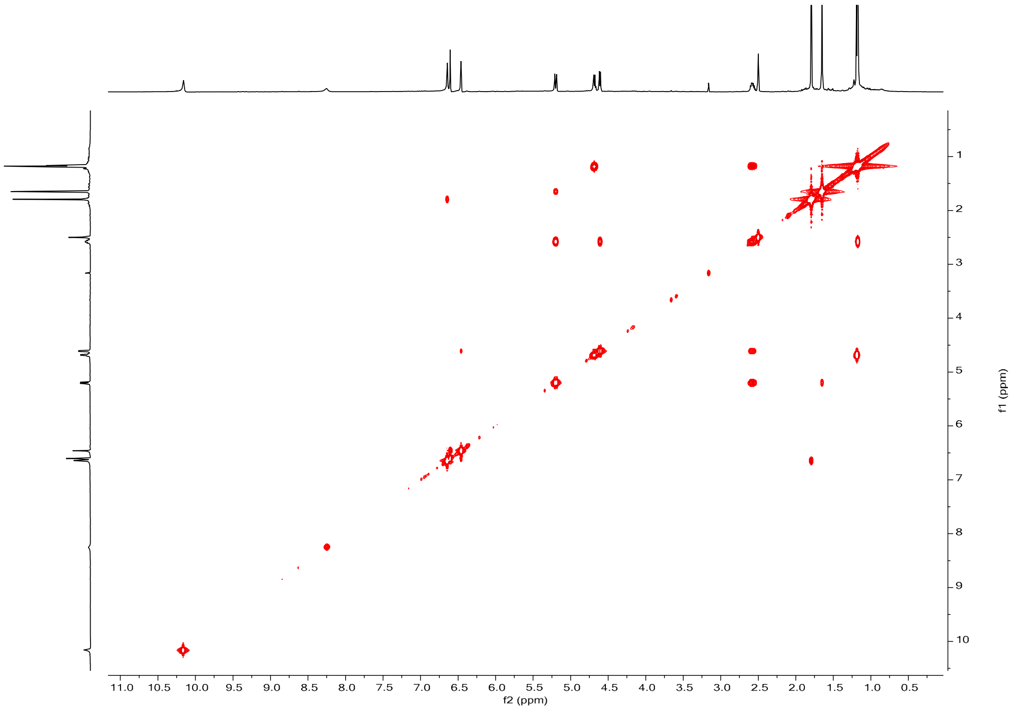


**Figure S40.** COSY spectrum of shengliangmycin B (**4**) in DMSO-$d_6$.

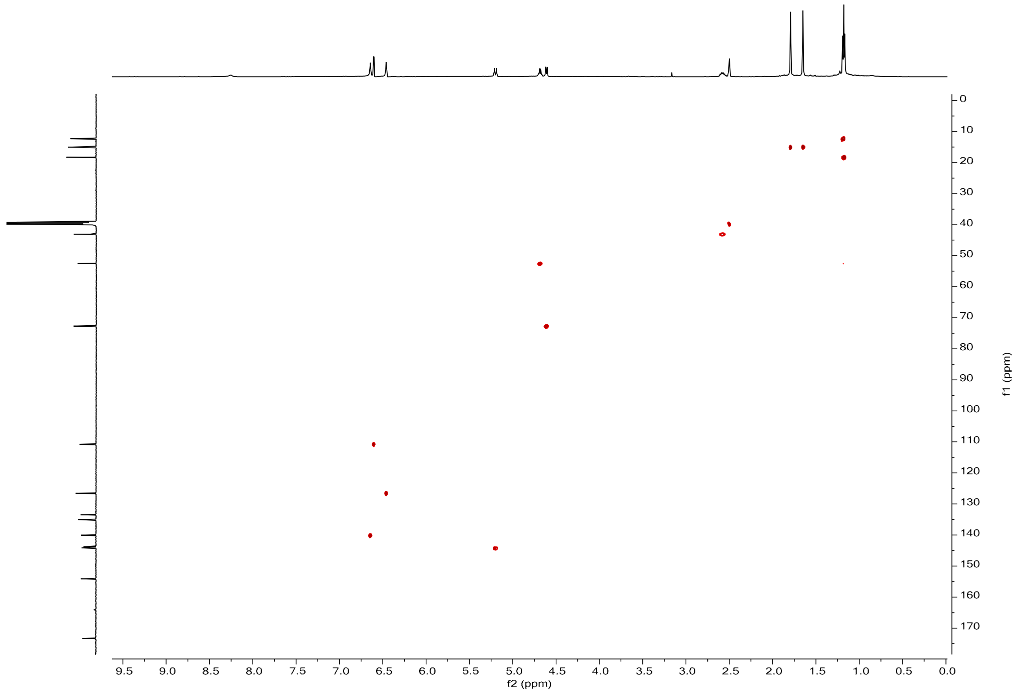


**Figure S41.** HSQC spectrum of shengliangmycin B (**4**) in DMSO-$d_6$.

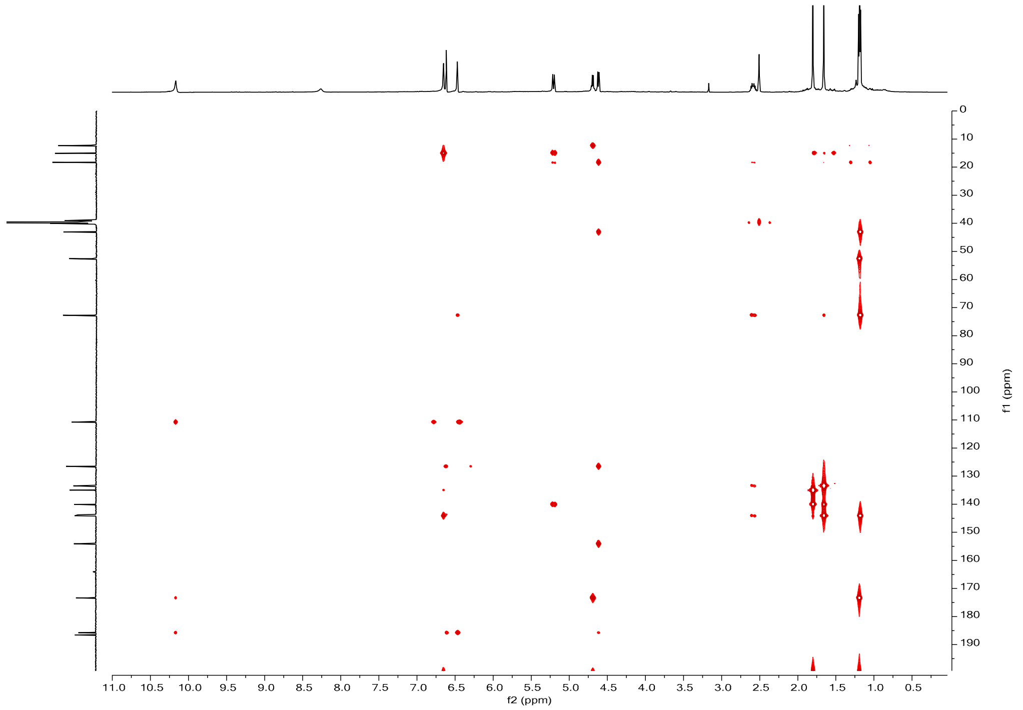


**Figure S42.** HMBC spectrum of shengliangmycin B (**4**) in DMSO-$d_6$.

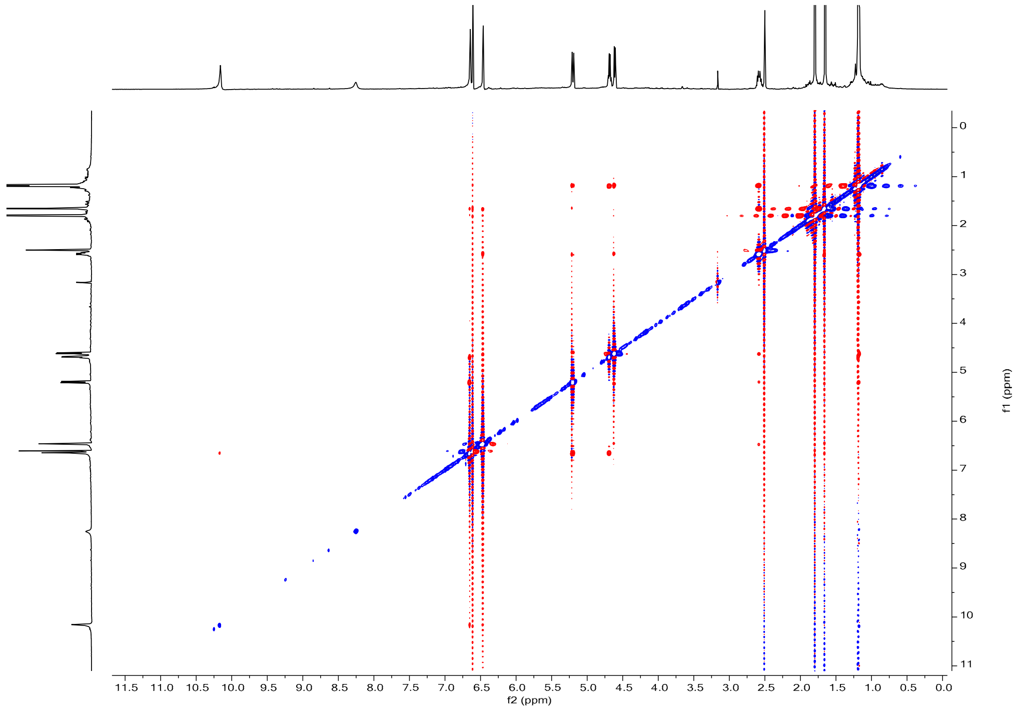

**Figure S43.** ROESY spectrum of shengliangmycin B (**4**) in DMSO-$d_6$.

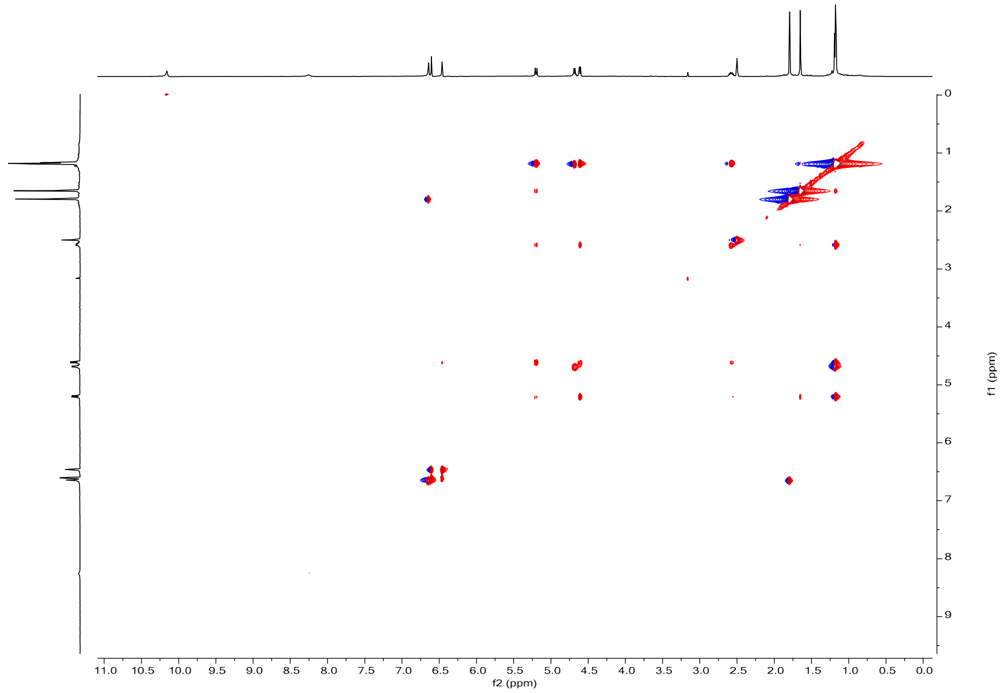

**Figure S44.** TOCSY spectrum of shengliangmycin B (**4**) in DMSO-$d_6$.

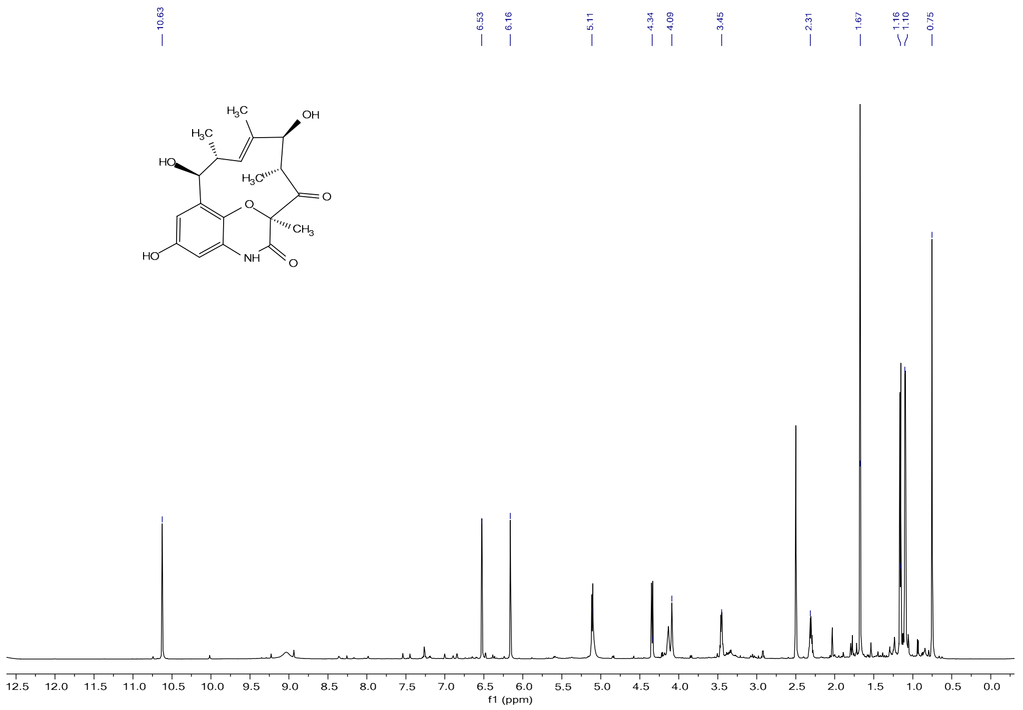


**Figure S45.** $^1$H-NMR spectrum of shengliangmycin D (**5**) at 700 MHz in DMSO-$d_6$.

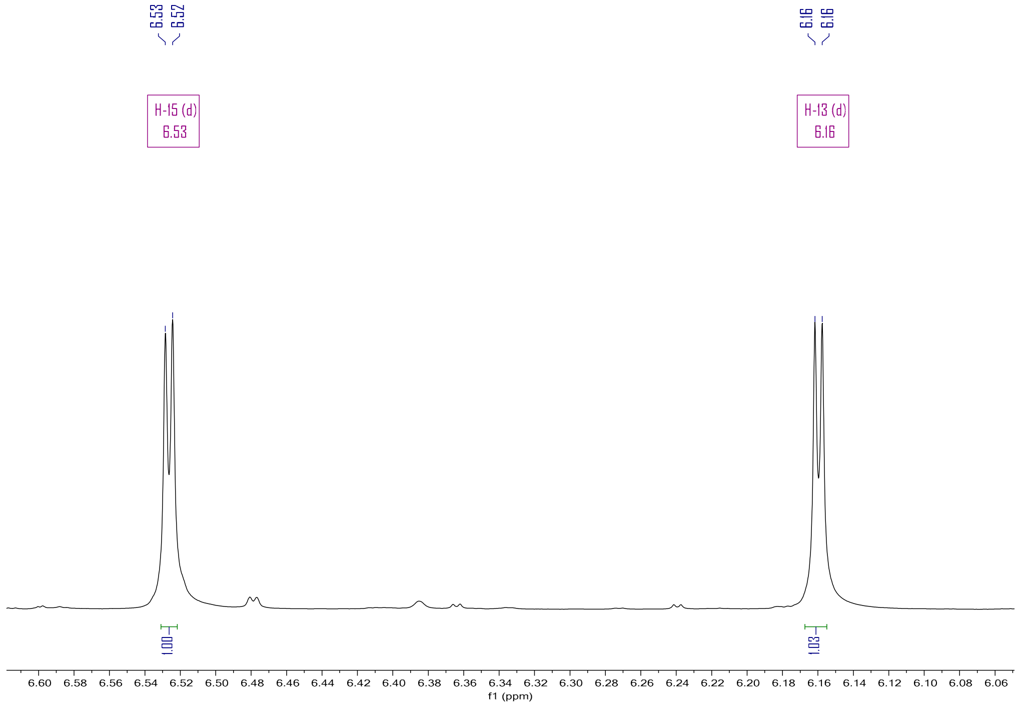


**Figure S46.** Expanded $^1$H-NMR spectrum ($\delta_H$ 6.6–6.0) of shengliangmycin D (**5**) at 700 MHz in DMSO-$d_6$.

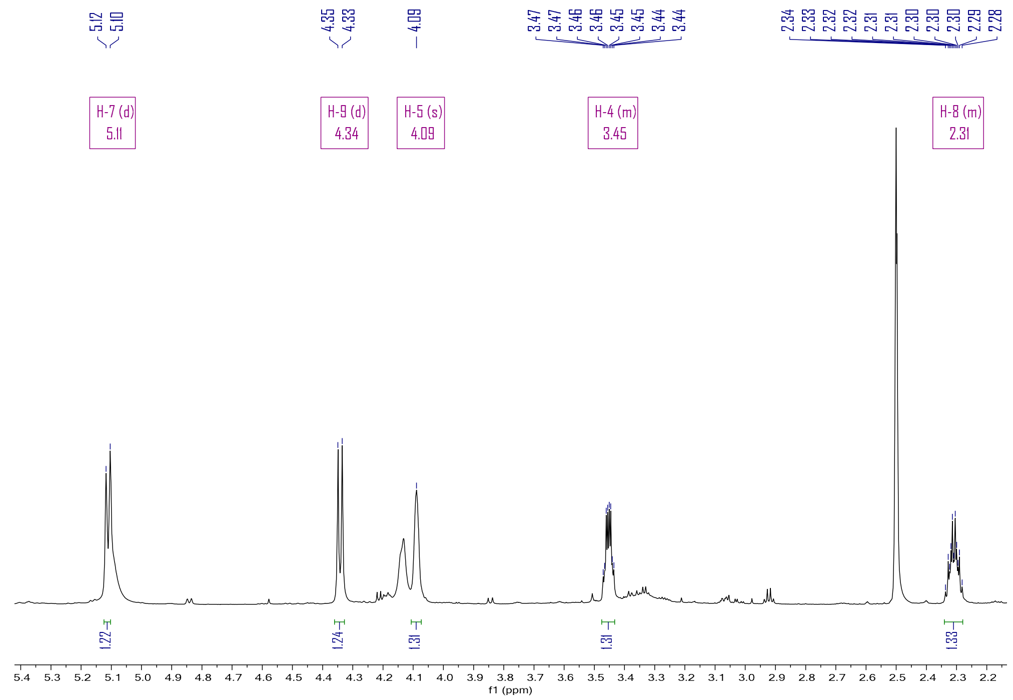


**Figure S47.** Expanded $^{1}$H-NMR spectrum ($\delta_H$ 5.4–2.2) of shengliangmycin D (**5**) at 700 MHz in DMSO-*d*$_6$.

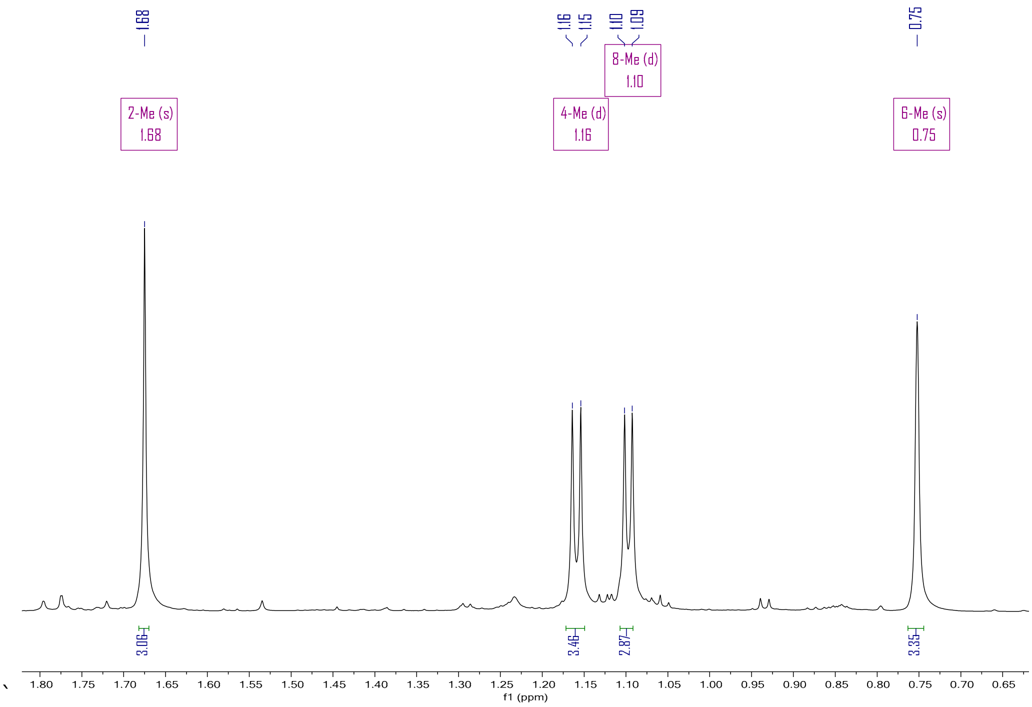


**Figure S48.** Expanded $^{1}$H-NMR spectrum ($\delta_H$ 1.8–0.6) of shengliangmycin D (**5**) at 700 MHz in DMSO-*d*$_6$.

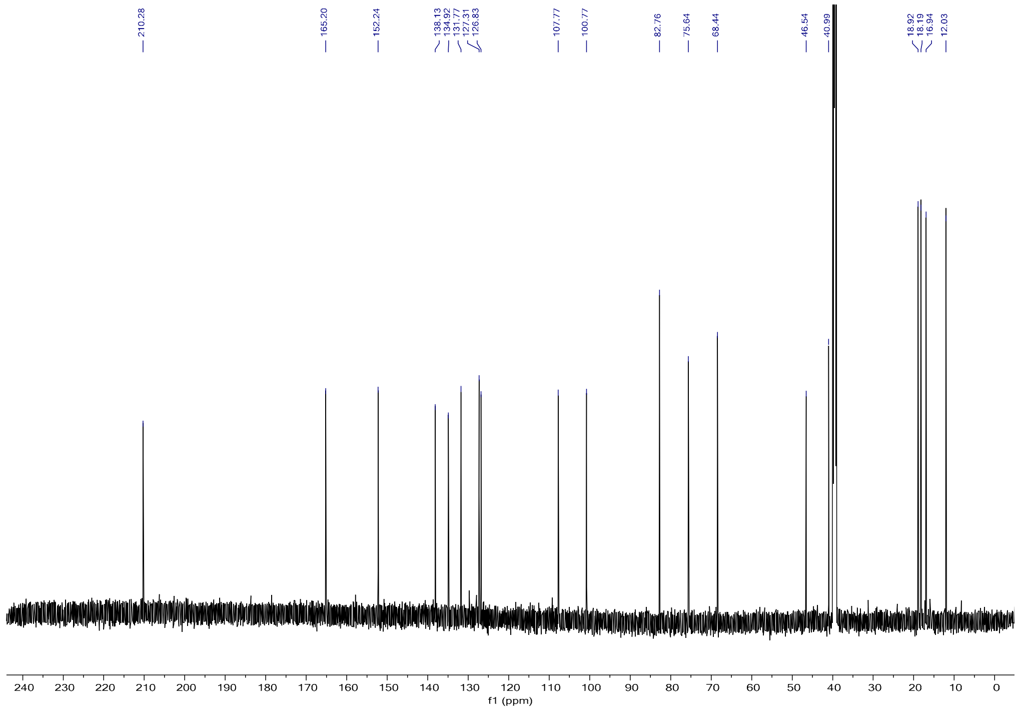


**Figure S49.** $^{13}$C-NMR spectrum of shengliangmycin D (**5**) at 175 MHz in DMSO-$d_6$.

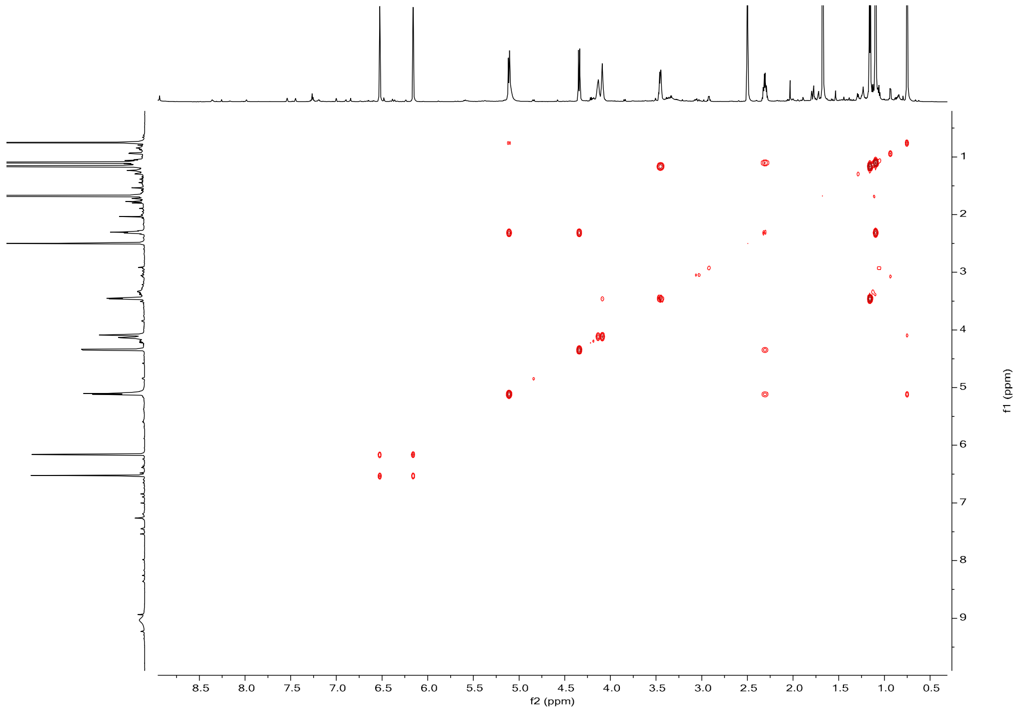


**Figure S50.** COSY spectrum of shengliangmycin D (**5**) in DMSO-$d_6$.

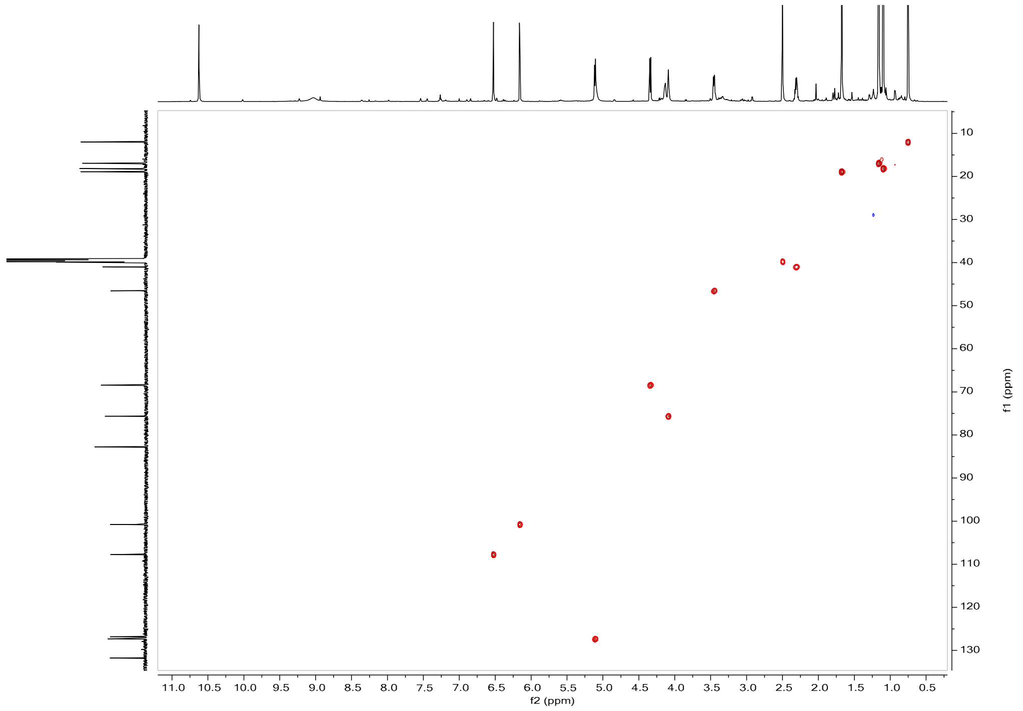


**Figure S51.** HSQC spectrum of shengliangmycin D (**5**) in DMSO-$d_6$.

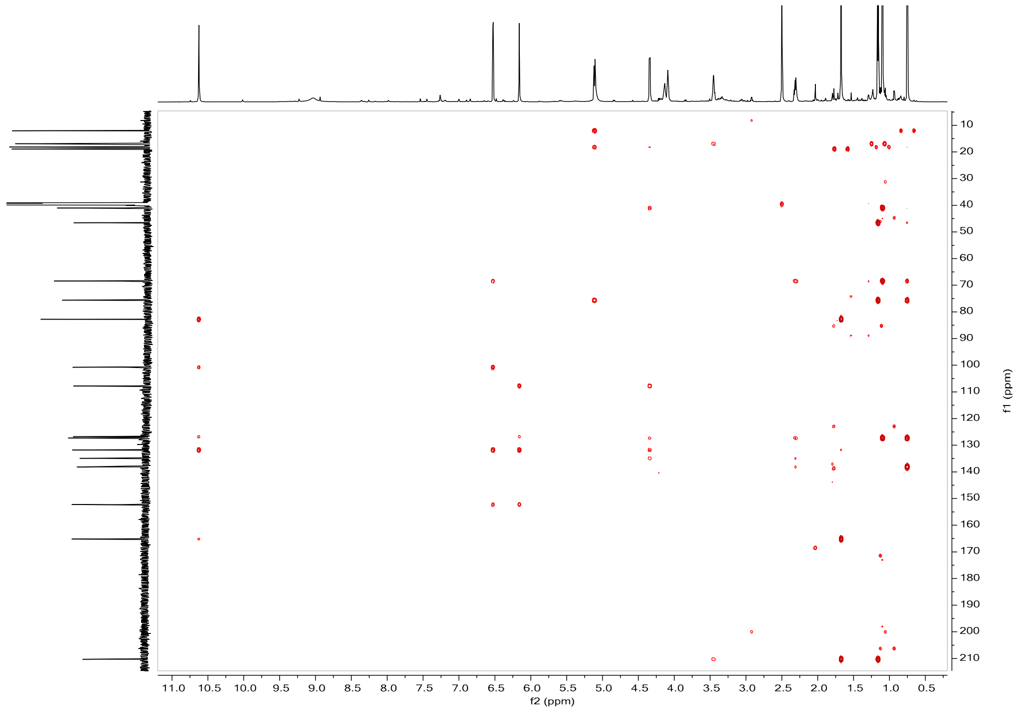


**Figure S52.** HMBC spectrum of shengliangmycin D (**5**) in DMSO-$d_6$.

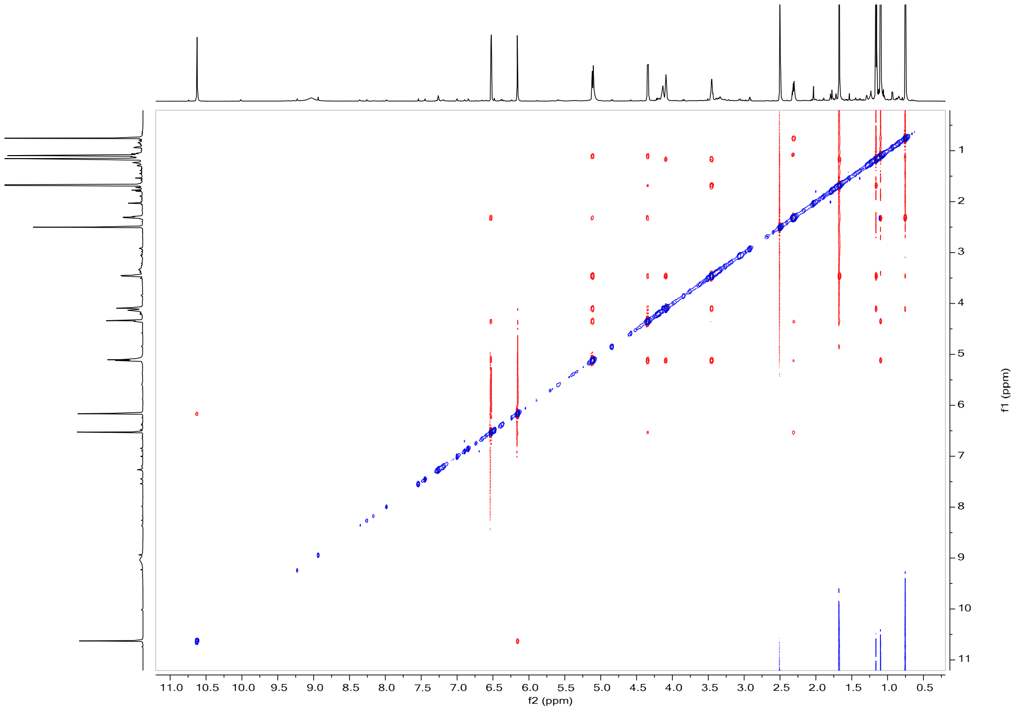


**Figure S53.** ROESY spectrum of shengliangmycin D (**5**) in DMSO-$d_6$.

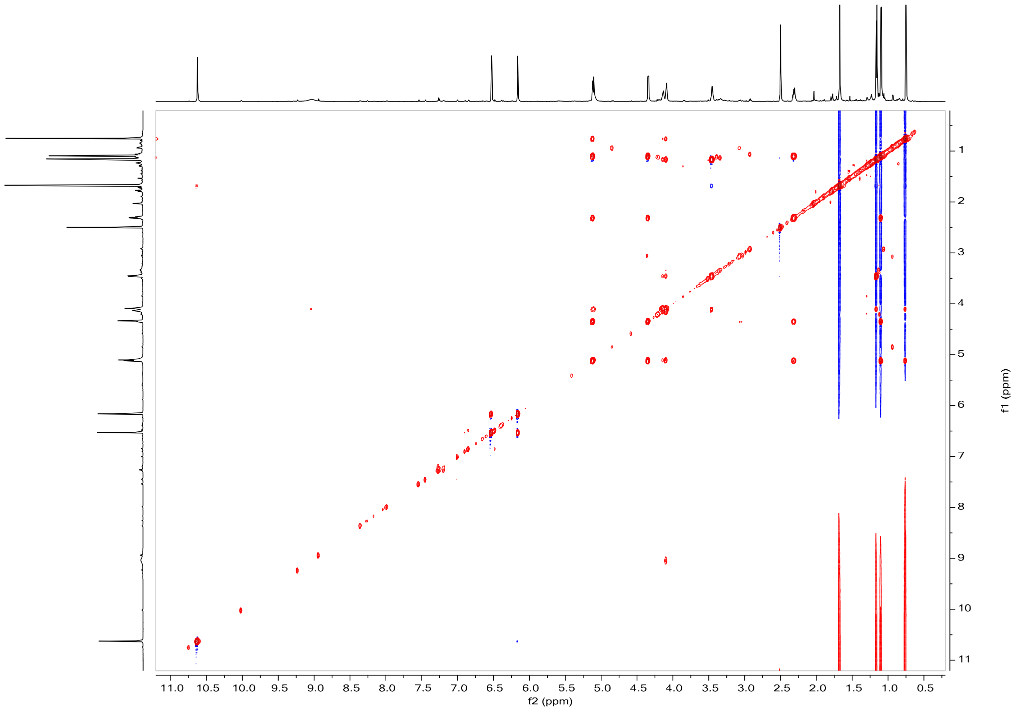


**Figure S54.** TOCSY spectrum of shengliangmycin D (**5**) in DMSO-$d_6$.

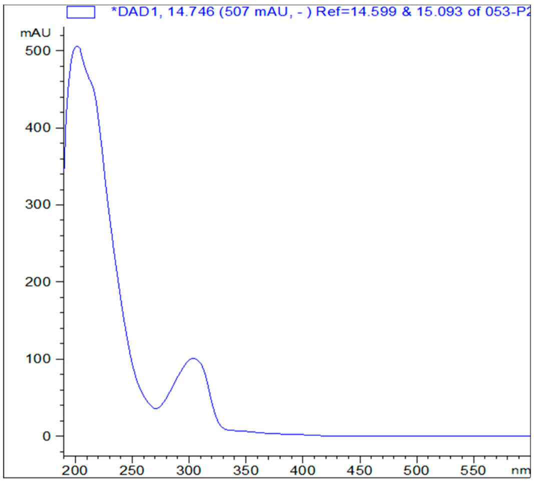


cebulactam A3 (**1**)

**Figure S55.** UV spectrum of cebulactam A3 (**1**).

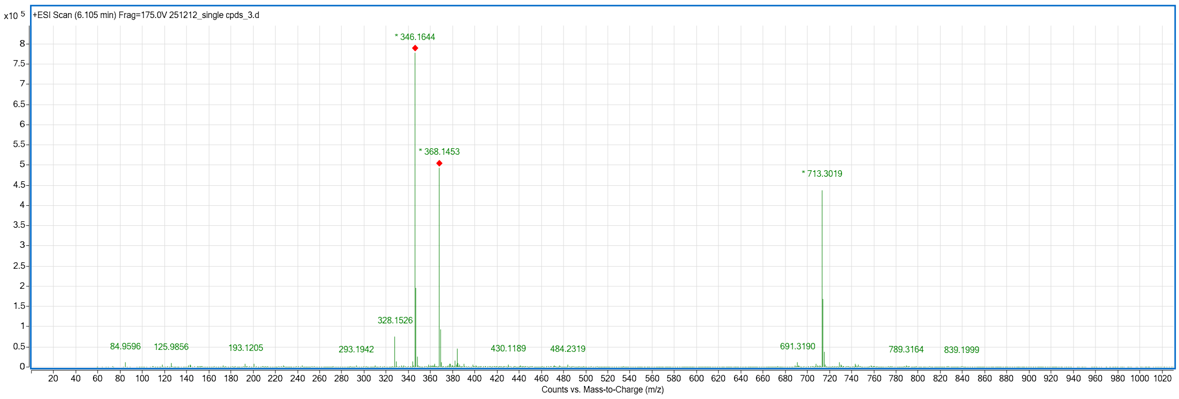


**Figure S56.** HR-ESI-MS data of cebulactam A3 (**1**).

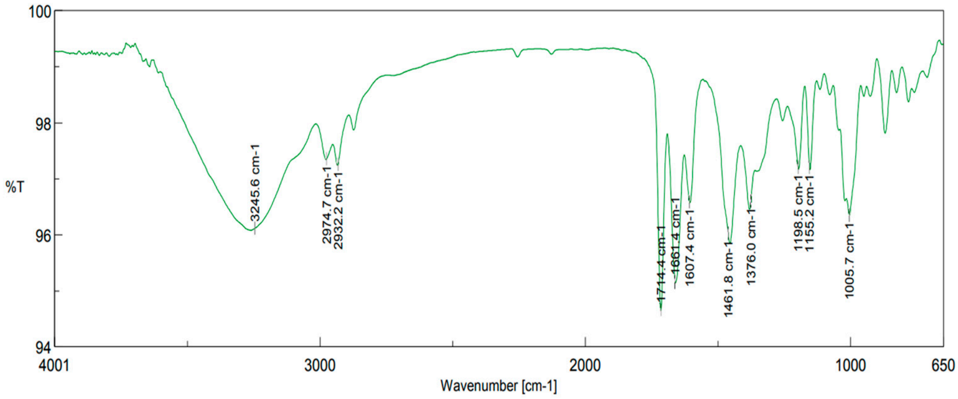


**Figure S57.** FT-IR spectrum of cebulactam A3 (**1**).

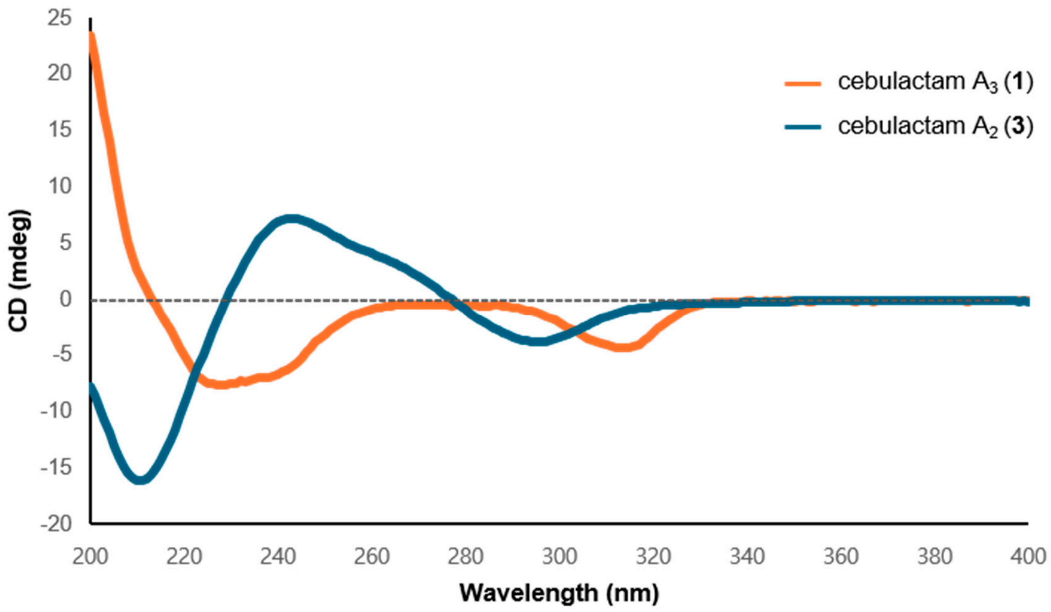


**Figure S58.** The circular dichroism (CD) spectra of cebulactams $A_3$ and $A_2$ (**1** and **3**) at a concentration of 0.1 mg/mL in MeOH.

**A**

| Sequencing Primer Name Primer Sequences | PCR Primer Name Primer Sequences |
|---|---|
| 785F 5' (GGA TTA GAT ACC CTG GTA) 3' | 27F 5' (AGA GTT TGA TCM TGG CTC AG) 3 |
| 907R 5' (CCG TCA ATT CMT TTR AGT TT) 3' | 1492R 5' (TAC GGY TAC CTT GTT ACG ACT T) 3' |

**B**

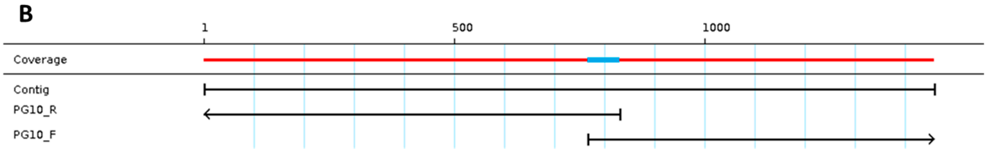


**C**

| Subject | | | | | | Score | | Identities | |
|---|---|---|---|---|---|---|---|---|---|
| Accession | Description | Length | Start | End | Coverage | Bit | E-Value | Match/Total | Pct.(%) |
| NR_044047.1 | Saccharopolyspora cebuensis | 1483 | 21 | 1472 | 97 | 2660 | 0.0 | 1448/1452 | 99.72 |

| Kingdom | Family | Genus | Species |
|---|---|---|---|
| Bacteria | Pseudonocardiaceae | Saccharopolyspora | Saccharopolyspora cebuensis |

**Figure S59.** 16S rRNA gene sequence data of PG10. (A) Primer information of 16S rRNA gene sequencing, (B) Contig assembly and overlap of sequencing reads (for details, see **Supplementary note**), (C) identification result from the 16S rRNA gene sequencing.

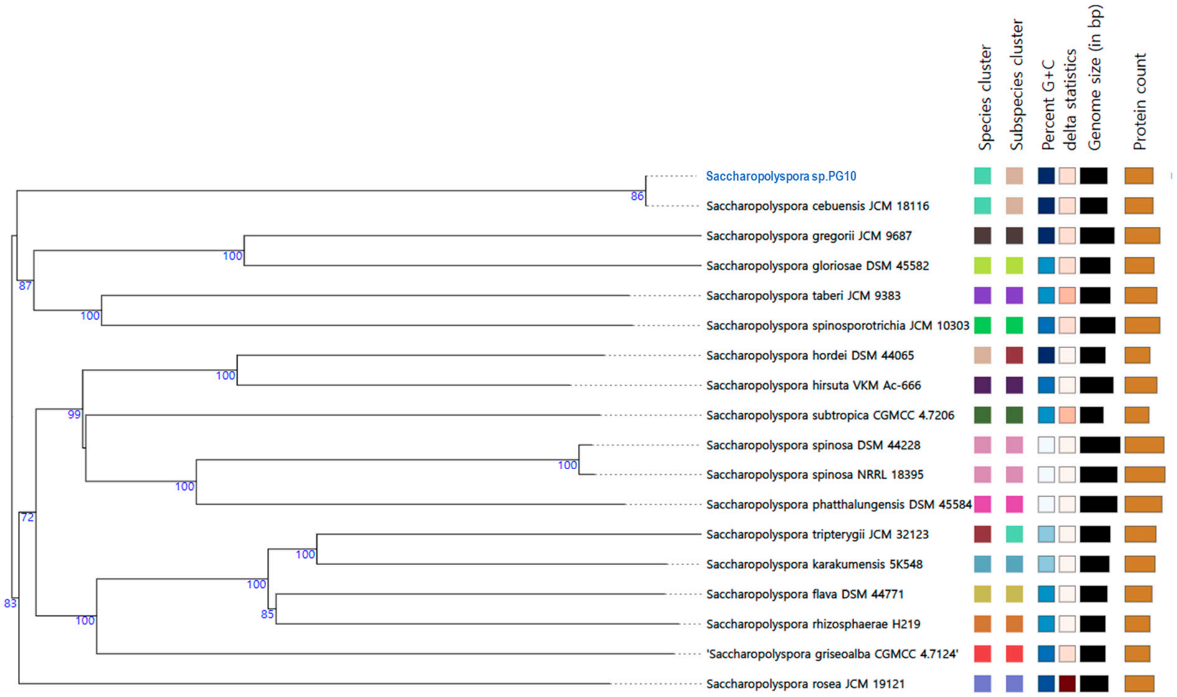


**Figure S60.** Whole-genome-based phylogenetic tree of PG10.

**Table S1**. $^{1}$H and $^{13}$C NMR spectroscopic data of **2–5** in DMSO-$d_6$.

| Position | cebulactam A$_1$ (**2**) | | cebulactam A$_2$ (**3**) | | shengliangmycin B (**4**) | | shengliangmycin D (**5**) | |
|---|---|---|---|---|---|---|---|---|
| | $\delta_C$, Type | $\delta_H$, Mult (*J* in Hz) | $\delta_C$, Type | $\delta_H$, Mult (*J* in Hz) | $\delta_C$, Type | $\delta_H$, Mult (*J* in Hz) | $\delta_C$, Type | $\delta_H$, Mult (*J* in Hz) |
| NH | | 8.94, s | | 9.36, s | | 10.16, s | | 10.63, s |
| 1 | 171.5, C | | 172.5, C | | 173.4, C | | 165.2, C | |
| 2 | 46.1, CH | 3.34, q (7.0) | 53.1, CH | 3.88, m | 52.5, CH | 4.68, q (6.5) | 82.8, C | |
| 2-Me | 15.9, $CH_3$ | 1.13, d (7.0) | 14.1, $CH_3$ | 1.16, d (7.0) | 12.3, $CH_3$ | 1.18, d (6.5) | 18.9, $CH_3$ | 1.67, s |
| 3 | 206.3, C | | 205.1, C | | 200.0, C | | 210.3, C | |
| 4 | 44.7, CH | 3.07, m | 46.3, CH | 3.25, m | 135.0, C | | 46.5, CH | 3.45, m |
| 4-Me | 17.2, $CH_3$ | 0.93, d (7.0) | 16.2, $CH_3$ | 0.92, d (7.0) | 15.1, $CH_3$ | 1.79, s | 16.9, $CH_3$ | 1.16, d (7.0) |
| 5 | 122.9, CH | 4.84, d (10.0) | 123.3, CH | 4.92, dd (10.0, 1.5) | 140.1, CH | 6.64, s | 75.6, CH | 4.09, s |
| 6 | 138.7, C | | 143.2, C | | 133.5, C | | 138.1, C | |
| 6-Me | 18.3, $CH_3$ | 1.78, s | 19.0, $CH_3$ | 1.92, d (1.5) | 15.0, $CH_3$ | 1.65, s | 12.0, $CH_3$ | 0.75, s |
| 7 | 85.3, CH | 4.21, d (8.0) | 84.1, CH | 4.18, d (10.0) | 144.2, CH | 5.20, d (11.0) | 127.3, CH | 5.11, d (9.0) |
| 8 | 40.4, CH | 1.68, m | 44.0, CH | 1.33, m | 43.1, CH | 2.58, m | 41.0, CH | 2.31, m |
| 8-Me | 16.0,$CH_3$ | 1.11, d (6.5) | 14.3, $CH_3$ | 1.04, d (6.5) | 18.3, $CH_3$ | 1.17, d (6.0) | 18.2, $CH_3$ | 1.10, d (6.5) |
| 9 | 68.8, CH | 4.19, d (10.0) | 68.8, CH | 4.19, m | 72.7, CH | 4.61, dd (8.0, 1.0) | 68.4, CH | 4.34, d (10.0) |
| 10 | 139.2, C | | 136.8, C | | 154.1, C | | 134.9, C | |
| 11 | 140.4, C | | 139.1, C | | 185.7, C | | 131.8, C | |
| 12 | 129.2, C | | 128.2, C | | 143.7, C | | 126.9, C | |
| 13 | 112.6, CH | 6.48, d (3.0) | 111.1, CH | 6.51, d (3.0) | 110.8, CH | 6.61, d (3.0) | 100.8, CH | 6.16, d (3.0) |
| 14 | 152.7, C | | 151.5, C | | 186.6, C | | 152.3, C | |
| 15 | 109.7, CH | 6.84, d (3.0) | 106.8, CH | 6.62, dd (3.0, 1.0) | 126.5, CH | 6.46, dd (3.0, 1.0) | 107.8, CH | 6.53, d (3.0) |

$^{1}$H and $^{13}$C data were recorded at 700 and 175 MHz, respectively.

**Table S2.** Isolated strains from diverse media.

| Strain | Medium | Antibiotics set |
|---|---|---|
| PG1 | TWYE | A |
| PG2 | SIM | A |
| PG3 | ISP5 | A |
| PG4 | GS | A |
| PG5 | SIM | A |
| PG6 | SIM | A |
| PG7 | SIM | A |
| PG8 | SIM | A |
| PG9 | TWYE | A |
| PG10 | TWYE | A |
| PG11 | GS | A |
| PG12 | ISP5 | A |
| PG13 | ISP5 | B |
| PG14 | SIM | A |
| PG15 | TWYE | B |
| PG16 | TWYE | B |
| PG17 | TWYE | A |
| PG18 | TWYE | A |
| PG19 | TWYE | A |
| PG20 | GS | B |
| PG21 | ISP5 | A |

**Table S3.** Predicted functions of CDSs in the cebulactam gene cluster based on whole-genome sequencing data.

| CDS | Amino acids | Proposed function | %Identity /similarity | Accession number |
|---|---|---|---|---|
| ctg1_2166 | 546 | ABC transporter substrate-binding protein | 100/100 | WP_345363726.1 |
| ctg1_2167 | 311 | ABC transporter permease | 100/100 | WP_345363727.1 |
| ctg1_2168 | 276 | ABC transporter permease | 100/100 | WP_345363728.1 |
| ctg1_2169 | 520 | ABC transporter ATP-binding protein | 100/100 | WP_345363729.1 |
| ctg1_2170 | 406 | acyl-CoA dehydrogenase family protein | 100/100 | WP_345363730.1 |
| ctg1_2171 | 332 | MsnO8 family LLM class oxidoreductase | 100/100 | WP_345363732.1 |
| ctg1_2172 | 483 | MFS transporter | 100/100 | WP_345363734.1 |
| ctg1_2173 | 916 | helix-turn-helix transcriptional regulator | 100/100 | WP_345363736.1 |
| ctg1_2174 | 127 | DoxX family protein | 100/100 | WP_345363738.1 |
| ctg1_2175 | 292 | ROK family protein | 100/100 | WP_345363740.1 |
| ctg1_2176 | 144 | type II 3-dehydroquinate dehydratase | 100/100 | WP_345363742.1 |
| ctg1_2177 | 387 | DegT/DnrJ/EryC1/StrS family aminotransferase | 90/94 | WP_150066298.1 |
| ctg1_2178 | 364 | Gfo/Idh/MocA family protein | 100/100 | WP_345363746.1 |
| ctg1_2179 | 228 | HAD-IA family hydrolase | 100/100 | WP_345363748.1 |
| ctg1_2180 | 344 | 3-dehydroquinate synthase family protein | 100/100 | WP_345363750.1 |
| ctg1_2181 | 267 | hypothetical protein | 99/100 | WP_345363752.1 |
| ctg1_2182 | 4491 | SDR family NAD(P)-dependent oxidoreductase | 100/100 | WP_345363754.1 |
| ctg1_2183 | 1801 | type I polyketide synthase | 99/100 | WP_425570099.1 |
| ctg1_2184 | 1033 | type I polyketide synthase | 100/100 | MEY8040927.1 |
| ctg1_2185 | 257 | arylamine N-acetyltransferase family protein | 100/100 | WP_345363760.1 |
| ctg1_2186 | 540 | bifunctional 3-(3-hydroxy-phenyl)propionate/3-hydroxycinnamic acid hydroxylase MhpA | 98/98 | WP_433872808.1 |
| ctg1_2187 | 423 | 3-deoxy-7-phosphoheptulonate synthase class II | 100/100 | WP_345363764.1 |
| ctg1_2188 | 95 | hypothetical protein | 100/100 | WP_345363766.1 |
| ctg1_2189 | 133 | ChaB family protein | 100/100 | WP_345363768.1 |
| ctg1_2190 | 234 | GAF and ANTAR domain-containing protein | 100/100 | WP_345363770.1 |
| ctg1_2191 | 178 | SdrD B-like domain-containing protein | 100/100 | WP_345363772.1 |
| ctg1_2192 | 237 | zinc ABC transporter ATP-binding protein AztA | 100/100 | WP_345363774.1 |
| ctg1_2193 | 298 | zinc ABC transporter permease AztB | 100/100 | WP_345363776.1 |
| ctg1_2194 | 391 | WD40 repeat domain-containing protein | 100/100 | WP_345363778.1 |
| ctg1_2195 | 306 | zinc ABC transporter substrate-binding protein AztC | 100/100 | WP_345363780.1 |
| ctg1_2196 | 393 | zinc metallochaperone AztD | 99/100 | WP_345363782.1 |
| ctg1_2197 | 54 | NAD(P)/FAD-dependent oxidoreductase | 96/100 | WP_433872834.1 |
| ctg1_2198 | 342 | FAD-dependent oxidoreductase | 100/100 | GAA4873761.1 |
| ctg1_2199 | 66 | ferredoxin | 100/100 | WP_345363784.1 |
| ctg1_2200 | 235 | TetR/AcrR family transcriptional regulator | 100/100 | WP_345363786.1 |
| ctg1_2201 | 405 | cytochrome P450 | 99/100 | WP_345363788.1 |
| ctg1_2202 | 203 | TetR/AcrR family transcriptional regulator | 100/100 | WP_345363790.1 |
| ctg1_2203 | 447 | phenylacetate--CoA ligase PaaK | 100/100 | WP_345363792.1 |
| ctg1_2204 | 682 | phenylacetic acid degradation bifunctional protein PaaZ | 100/100 | WP_345363794.1 |
| ctg1_2205 | 313 | 1,2-phenylacetyl-CoA epoxidase subunit PaaA | 100/100 | WP_345363796.1 |

**Table S4.** Growth-inhibitory activities ($IC_{50}$) of compounds **1**–**5** against the tested fungal and bacterial strains.

| Compound | $IC_{50}$ (μM) | | | | |
|---|---|---|---|---|---|
| | Fungus | Gram Positive Bacteria | Gram Negative Bacteria | | |
| | *C. albicans* KCTC 7965 | *B. subtilis* ATCC 6051 | *P. aeruginosa* KCTC 22073 | *E. coli* ATCC 11775 | *Er. rhapontici* ATCC 29283 |
| **1** | >500 | >500 | 203.07 | >500 | >500 |
| **2** | >500 | >500 | 200.50 | >500 | >500 |
| **3** | >500 | 109.67 | 218.98 | >500 | >500 |
| **4** | >500 | 14.10 | 3.37 | >500 | >500 |
| **5** | >500 | >500 | >500 | >500 | >500 |
| gentamicin | - | <0.84 | <0.84 | <0.84 | <0.84 |
| cycloheximide | <1.42 | - | - | - | - |

**Table S5.** Isolation and cultivation media.

| Media | Components |
|---|---|
| **Isolation media** (33.0 g sea salt added; except Marine) | |
| SIM | 0.3 g casein, 1.0 g starch, 0.5 g $KNO_3$, 0.2 g $K_2HPO_4$, 0.1 g $MgSO_4 \cdot 7H_2O$, 0.1 g $CaCO_3$,1.0 L distilled water, 18.0 g agar powder |
| ISP5 | 1.0 g L-asparagine(anhydrous), 10.0 g glycerol, 1.0 g $K_2HPO_4$, 1.0 mg $FeSO_4 \cdot 7H_2O$, 1.0 mg $MnCl_2 \cdot 4H_2O$, 1.0 mg $ZnSO_4 \cdot 7H_2O$, 1.0L distilled water, 18.0 g agar powder |
| SCNA | 10.0 g starch, 0.3 g casein, 2.0g $KNO_3$, 1.0 L distilled water, 18.0 g agar powder |
| TWYE | 0.25 g yeast extract, 0.5 g $K_2HPO_4$, 1.0 L tap water, 18.0 g agar powder |
| GS | 20.0 g starch, 1.0 g $KNO_3$, 0.5 g $K_2HPO_4$, 0.5 g NaCl, 0.5 g $MgSO_4 \cdot 7H_2O$, 0.01 g $FeSO_4$, 1.0 L distilled water, 18.0 g agar powder |
| **Cultivation media** (33.0 g sea salt added; except R4) | |
| TSBY | 17.0 g tryptone, 3.0 g soytone, 2.5 g glucose, 5.0 g sodium chloride, 2.5 g dipotassium phosphate, 3.0 g yeast, 1.0 L distilled water |
| R4 | 5.0 g glucose, 1.0 g yeast extract, 5.0 g $MgCl_2 \cdot 6H_2O$, 2.0 g $CaCl_2 \cdot 2H_2O$, 1.5 g proline, 1.2 g valine, 3.0 g TES, 50.0 mg casamino acid, 100 mg $K_2SO_4$, 20.0 g sea salt, 1 mL trace element solution*, 1.0 L distilled water |
| | *trace element solution: 40 mg $ZnCl_2$, 200 mg $FeCl_3 \cdot 6H_2O$, 10 mg $CuCl_2 \cdot 2H_2O$, 10 mg $MnCl_2 \cdot 4H_2O$, $Na_2B_4O_7$, 10 mg $(NH_4)_6Mo_7O_{24} \cdot 4H_2O$, 1.0 L distilled water |

**Supplementary note.**

16S rRNA sequence of PG10 (overlapping region highlighted in blue)

ACGAACGCTGGCGGCGTGCTTAACACATGCAAGTCGAACGCTGAAGCATCTTCGGGTGTGGATGA
GTGGCGAACGGGTGAGTAACACGTGGGTAATCTGCCCTGCACTCTGGGATAAGCCCTGGAAACG
GGGTCTAATACCGGATAGGACATTCTGCCGCATGGTGGGGTGTGGAAAGTTCCGGCGGTGCAGGA
TGAGCCCGCGGCCTATCAGCTTGTTGGTGGGGTGATGGCCTACCAAGGCGACGACGGGTAGCCGG
CCTGAGAGGGTGACCGGCCACACTGGGACTGAGACACGGCCCAGACTCCTACGGGAGGCAGCA
GTGGGGAATCTTGCGCAATGGGCGAAAGCCTGACGCAGCAACGCCGCGTGGGGGATGACGGCCT
TCGGGTTGTAAACCTCTTTCGACAGGGACGAAGCCTTCGGGTGACGGTACCTGTAGAAGAAGCAC
CGGCTAACTACGTGCCAGCAGCCGCGGTAATACGTAGGGTGCGAGCGTTGTCCGGATTTATTGGG
CGTAAAGAGCTCGTAGGCGGTTTGTCGCGTCGGCCGTGAAAACCTGCAGCTTAACTGTGGGCGTG
CGGTCGATACGGGCAGACTTGAGTTCGGCAGGGGAGACTGGAATTCCTGGTGTAGCGGTGAAATG
CGCAGATATCAGGAGGAACACCGGTGGCGAAGGCGGGTCTCTGGGCCGATACTGACGCTGAGGA
GCGAAAGCGTGGGGAGCGAACAGGATTAGATACCCTGGTAGTCCACGCCGTAAACGTTGGGCGC
TAGGTGTGGGGACCGGTTCCACGGTTTCTGTGCCGTAGCTAACGCATTAAGCGCCCCGCCTGGGG
AGTACGGCCGCAAGGCTAAAACTCAAAGGAATTGACGGGGGCCCGCACAAGCGGCGGAGCATG
TGGATTAATTCGATGCAACGCGAAGAACCTTACCTGGGTTTGACATGCACCAGATTGCCCCTGAG
AGGGGGTTTCCCTTGTGGTTGGTGTACAGGTGGTGCATGGCTGTCGTCAGCTCGTGTCGTGAGATG
TTGGGTTAAGTCCCGCAACGAGCGCAACCCTTGTCCTGTGTTGCCAGCACGTAATGGTGGGGACT
CGCGGGAGACTGCCGGGGTCAACTCGGAGGAAGGTGGGGATGACGTCAAGTCATCATGCCCCTT
ATGCCCAGGGCTTCACACATGCTACAATGGCTGGTACAGAGGGTGGCGATACCGTGAGGTGGAGC
GAATCCCTTAAAGCCGGTCTCAGTTCGGATCGGGGTCTGCAACTCGACCCCGTGAAGTCGGAGTC
GCTAGTAATCGCAGATCAGCAGTGCTGCGGTGAATACGTTCCCGGGCCTTGTACACACCGCCCGT
CACGTCATGAAAGTCGGTAACACCCGAAGCCCATGGCCTAACCCTGTTGGGGTGGAGTGGTCGA
AGGTGGGACTGGCGATTGGGACGAAGTCGTACCAGGG
